# Dissipative Particle Dynamics Models of Encapsulated Microbubbles and Nanoscale Gas Vesicles for Biomedical Ultrasound Simulations

**DOI:** 10.1021/acsanm.5c02783

**Published:** 2025-08-04

**Authors:** Nikolaos Ntarakas, Maša Lah, Daniel Svenšek, Tilen Potisk, Matej Praprotnik

**Affiliations:** † Laboratory for Molecular Modeling, National Institute of Chemistry, Hajdrihova 19, SI-1001 Ljubljana, Slovenia; ‡ Department of Physics, Faculty of Mathematics and Physics, University of Ljubljana, Jadranska 19, SI-1000 Ljubljana, Slovenia; § Universitat de Barcelona Institute of Complex Systems, C/Marti i Franques 1, 08028 Barcelona, Spain

**Keywords:** ultrasound, gas vesicles, proteinaceous nanostructures, microbubbles, particle simulations, mesoscopic modeling

## Abstract

Ultrasound-guided drug and gene delivery (usdg) enables controlled and spatially precise delivery of drugs and macromolecules, encapsulated in microbubbles (embs) and nanoscale gas vesicles (gvs), to target areas such as cancer tumors. It is a noninvasive, high precision, low toxicity process with drastically reduced drug dosage. Rheological and acoustic properties of gvs and embs critically affect the outcome of usdg and imaging. Detailed understanding and modeling of their physical properties is thus essential for ultrasound-mediated therapeutic applications. State-of-the-art continuum models of shelled bodies cannot incorporate critical details such as varying thickness of the encapsulating shell or specific interactions between its constituents and interior or exterior solvents. Such modeling approaches also do not allow for detailed modeling of chemical surface functionalizations, which are crucial for tuning the gv–blood interactions. We develop a general particle-based modeling framework for encapsulated bodies that accurately captures elastic and rheological properties of gvs and embs at the mesoscopic and nanoscale levels. We use dissipative particle dynamics to model the solvent, the gaseous phase in the capsid, and the triangulated surfaces of immersed objects. Their elastic behavior is studied and validated through stretching and buckling simulations, eigenmode analysis, shear flow simulations, and comparison of predicted gv buckling pressure with published experimental data. The presented modeling approach paves the way for large-scale simulations of nanoscale and microscale encapsulated bodies of different shapes and local anisotropy, capturing their dynamics, interactions, and collective behavior.

## Graphical abstract

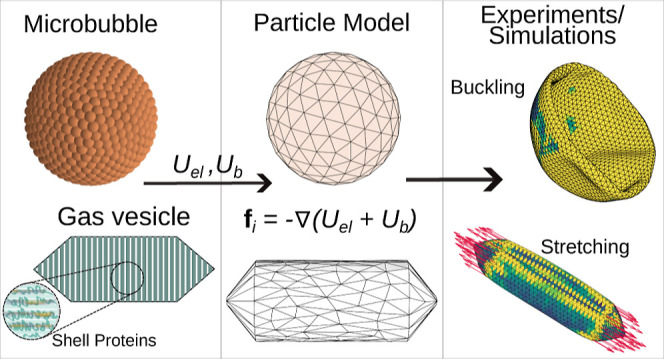

## 1 Introduction

Ultrasound (us) is increasingly being used in biomedical applications to diagnose many types of cancer, for blood flow analysis and therapeutic applications, including thermal tissue coagulation, kidney stones fragmentation, bone healing, mechanical tissue disruption and in cases of joint inflammation or rheumatoid arthritis.^1–7^ It offers numerous advantages, such as functionality in opaque media, relatively high spatial precision on the micrometer scale and fast, reconfigurable field formation.^8^ These features have made us a cornerstone of modern biomedical imaging and therapy.

To further enhance the capabilities of us in diagnostics and therapeutics, a diverse set of responsive agents has been developed, including encapsulated biomaterials and even synthetic nano- and microrobots.^9,10^ Encapsulated biomaterials^2,11^ have emerged as powerful tools due to their unique design and shell properties make them highly adaptable for two major us biomedical applications: enhancing us imaging as ultrasound contrast agents (ucas)^1,5,11,12^ and enabling the encapsulation and targeted delivery of therapeutic drugs.^1,2,11–18^ Among these materials, encapsulated microbubbles (embs) and gas vesicles (gvs) have garnered significant attention for their adaptability and effectiveness in such applications. Radial oscillations of embs and gvs generate strong nonlinear acoustic signals with a unique signature in the acoustic field and a frequency range much greater than that produced by tissues. This allows them to generate significant us contrast across a range of frequencies, supporting harmonic, multiplexed, and multimodal us imaging, as well as cell-specific molecular targeting.^19,20^

embs injected into the bloodstream are already being used for echocardiography,^11,13,21^ which is one of the essential tools for diagnosing cardiovascular diseases. embs are typically 1 μm in diameter and consist of biologically inert gases, such as air or gases with lower water solubility, stabilized within a lipid, protein, or polymer shell.^1,12,22^ When an emb is subjected to a high-intensity acoustic field, it expands in volume and collapses violently. This process is known as inertial cavitation.^23^ In contrast, during noninertial or stable cavitation, embs oscillate with relatively minor deformations under lower acoustic pressure amplitudes. Cavitation is utilized in sonoporation,^24^ which is a targeted drug delivery technique that creates temporary pores in cell membranes, enabling the entry of foreign substances.^14,25^ Exploiting the sonoporation effect for disease therapy has many advantages, for instance, injecting embs intravenously can lower drug dosage and minimize side effects of nonspecific drug delivery into healthy organs as the embs only collapse in specific diseased areas due to focused us irradiation. One limitation to the use of embs is their size, which prevents them from extravasating into tumors. To circumvent this limitation, gvs have been introduced as a new class of nanoscale us imaging agents.^26–32^

gvs are gas-filled, protein-shelled nanostructures produced by buoyant photosynthetic microbes. These vesicles vary in size, with widths ranging from 45 to 200 nm and lengths from 100 to 800 nm, depending on their genetic origin, and can withstand external pressures of several bar without collapsing. Recently, gvs have also been shown to increase the influx of calcium ions, when attached to biological cells and insonated by us.^33^ The structure of several types of gvs has already been characterized using cryo-EM (Cryogenic electron microscopy) and cryo-ET (Cryogenic electron tomography),^34,35^ revealing that the main structural protein GvpA self-assembles helically in a cylindrical shape, which closes off on both sides by cone-shaped tips. The polarity of the helical assembly inverts at the midpoint of the gv cylinder, which may act as an elongation center for growth. This implies that the ribs are oriented helically along the cylinder, reversing direction at the central rib.^34,36^ Unlike embs, which confine preloaded gas in an unstable state, gvs have 2 nm-thick protein shells that exclude water but allow gas to diffuse in and out of their interior.^37^

The acoustic behavior of embs and gvs is influenced by several factors, such as the viscosity and temperature of the surrounding fluid, the applied acoustic pressure and the physical characteristics of the objects, including size and shell properties like viscosity and elasticity.^38,39^ In addition, the presence of nearby vessel walls or cells can significantly affect the emb behavior.^39^ Unlike the detailed modeling of blood flow^40–55^ or cloud cavitation collapse,^56,57^ the current theoretical modeling of emb oscillations primarily relies on the continuum theory developed by Rayleigh and Plesset for a single, free, spherically symmetric bubble in an infinite liquid with constant viscosity.^58,59^ The Rayleigh-Plesset emb model incorporates several assumptions, including the ideal gas behavior of the encapsulated gas and the absence of a shell. A series of increasingly complex models have been developed to more accurately represent the dynamics of embs in vivo—particularly those excited by us while flowing through small blood vessels. Despite these improvements, the models continue to rely on various assumptions and simplifications.^60–62^ A novel stress–strain method^63^ was derived to characterize the viscoelastic shells of individual lipid-shelled us-driven microbubbles, aiming to extract their elastic and viscous properties with minimal assumptions.

Continuum models for gvs are very scarce, with the exception of finite element models for various types of gvs, such as the Anabaena flos-aquae^20,34,64,65^ and the ,^66^ which focus purely on mechanical properties in vacuum, without explicitly modeling the surrounding solvent or encapsulated gas. A microscopic model has been reported,^67^ in which a model for the GvpA rib was developed and used to calculate the Young’s moduli of the gv shell. A recent paper^68^ examined the use of gvs as cavitation nuclei, where the authors conducted simulations of bubbles formed by the coalescence of gas released from destroyed gvs. Although significant efforts have been made to improve continuum models for embs and gvs,^38,60,62,66,69–77^ accurately modeling the shell properties before and after insonation remains a challenging task. The applicability of continuum models in these scenarios is limited, mainly due to the lack of detailed interfacial constitutive models.^60^ These limitations of existing continuum models preclude an accurate description of cavitation, drastically degrading the prediction of drug delivery outcomes.

The development of novel embs and gvs models using mesoscopic particle-based approaches tailored to the specific shell material is crucial to study changes in the material upon deformation and its mechanical response to interaction with us. Importantly, the mechanical behavior of ucas differs significantly between water and blood, due to variations in viscosity, elasticity, and the complex interplay with surrounding embs and vesicles in the bloodstream. Incorporating these factors into simulations is essential for accurate predictions of their performance in real physiological environments. To accurately capture the rheological and acoustic properties, as well as the dynamics of embs and gvs, we propose mesoscopic particle-based models inspired by the network models of red blood cells (rbcs). The proposed models are designed to be general enough to accommodate a wide range of physical systems. Here, we use the dissipative particle dynamics (dpd) method, a state-of-the-art particle-based method for modeling colloidal suspensions, polymers, soft matter, and simple fluids. We model mechanical properties of embs and gvs, including their behavior in stretching and buckling experiments. Our predictions for the buckling pressure of gvs are compared to experimental measurements. Furthermore, we determine fundamental eigenmodes of embs and gvs. Finally, we study their rheological properties under shear flow and compare them with analytical expressions.

## 2 Methods

The simulations were carried out using Mirheo,^78^ a high-throughput simulation package, specifically designed and optimized for dpd simulations. While isotropic elastic forces are already implemented in Mirheo, we modified and extended its functionalities to include orthotropic elastic forces, the gas pressure contribution as well as the obmd used in nonequilibrium simulations.

### 2.1 Dissipative Particle Dynamics

The dpd method^79^ is a particle-based mesoscopic simulation technique that allows modeling of fluids and soft matter.^80,81^ A dpd system is represented by *N* particles, which interact through pairwise effective potentials and move according to Newton’s second law. In a dpd simulation, a particle represents a cluster of molecules. In our case, the dpd particle represents a large number of water or gas molecules.

The interparticle force **F**_*ij*_ = **F**_*ij*_^*C*^ + **F**_*ij*_^*D*^ + **F**_*ij*_^*R*^ exerted by bead *j* on bead *i* consists of conservative, dissipative, and random forces^82^

1 $$F_{ij}^{C}=a_{\alpha \beta }\omega_{C}\left(r_{ij}\right){\mathrm{r̂}}_{ij}$$

2 $$F_{ij}^{D}=-\gamma_{\alpha \beta }\omega_{D}\left(r_{ij}\right)\left(v_{ij}\cdot {\mathrm{r̂}}_{ij}\right){\mathrm{r̂}}_{ij}$$

3 $$F_{ij}^{R}=\sigma_{\alpha \beta }\omega_{R}\left(r_{ij}\right)\Theta_{ij}{\mathrm{r̂}}_{ij}$$

where *a*_αβ_ and γ_αβ_ represent the conservative and dissipative parameters for a bead pair of species α and β (water, gas, uca object), specified in Table 1, σ_αβ_ is the random force amplitude connected with the dissipative parameter γ_αβ_ (S4.1), ${\mathrm{r̂}}_{ij}=\frac{r_{i}-r_{j}}{\left|r_{i}-r_{j}\right|}$ is the normalized vector along the interparticle axis, **v**_*ij*_ = **v**_*i*_ – **v**_*j*_ the relative velocity of the two interacting particles, ω_*C*_, ω_*D*_, ω_*R*_ are the weight kernels described in S4.1, while Θ_*ij*_ = Θ_*ji*_ is a zero-mean random Gaussian variable with unit variance, uncorrelated between different pairs of particles *i* and *j*

4 $$\left\langle \Theta_{ij}\left(t\right)\Theta_{kl}\left(t^{\prime }\right)\right\rangle =\left(\delta_{ik}\delta_{jl}+\delta_{jk}\delta_{il}\right)\delta \left(t-t^{\prime }\right)$$

**Table 1:** Values of the dpd Parameters Used, Unless Stated Otherwise, for emb and gv Unit Sets^a^

dpd parameter	value (emb unit set)	value (gv unit set)
*a*_ww_	$100.0\;\frac{k_{B}T_{0}}{r_{c}}$	$100.0\;\frac{k_{B}T_{0}}{r_{c}}$
*a*_wg_, *a*_gg_, *a*_oo_, *a*_og_	$0.0\;\frac{k_{B}T_{0}}{r_{c}}$	$0.0\;\frac{k_{B}T_{0}}{r_{c}}$
*a*_ow_	$40\;\frac{k_{B}T_{0}}{r_{c}}$	$40\;\frac{k_{B}T_{0}}{r_{c}}$
γ_ww_	$3.5\;\frac{m}{\tau }$	$18.0\;\frac{m}{\tau }$
γ_gg_	$11.0\;\frac{m}{\tau }$	$12.0\;\frac{m}{\tau }$
γ_wg_	$0.0\;\frac{m}{\tau }$	$0.0\;\frac{m}{\tau }$
γ_ow_	$7.4\;\frac{m}{\tau }$	$19.5\;\frac{m}{\tau }$
γ_og_	$0.2\;\frac{m}{\tau }$	$0.5\;\frac{m}{\tau }$
ρ_w_, ρ_g_	3.0 *r*_c_^–3^	3.0 *r*_c_^–3^
*k*_ww_	0.25	0.125
*k*_gg_	0.25	0.0
*k*_*fsi*_	0.5	0.5

a The subscripts denote the different types of beads: (o)­bject, (w)­ater, (g)­as.

The equation of state of a dpd fluid is quadratic in the density

5 $$p=\rho k_{B}T_{0}+\alpha a_{ww}\rho^{2}$$

where α ≈ 0.100*r*_c_^4^ is a semiempirical prefactor.^83^

### 2.2 Membrane Modeling

We represent the gv and emb membranes as triangulated surfaces, where each vertex corresponds to a dpd bead (particle). Interactions between these membrane particles are derived from a discretized continuum elastic energy, as detailed in the Supporting Information.

For embs, we generate spherical meshes using icosahedral subdivision, resulting in a surface composed of 2562 vertices. For gvs, the structure is constructed by stacking concentric circles of particles in a staggered configuration, which are then smoothly tapered to form the conical end-caps, resulting in a mesh with 1404 vertices. This coarse-grained representation captures both the global geometry and local elastic properties of the membranes while remaining computationally efficient at the mesoscale.

### 2.3 Fundamental Scales

The solvent and encapsulated gas phases are modeled using dpd, which allows capturing relevant rheological properties, such as viscosity, on large spatiotemporal scales.^84^ To parametrize the interactions between the various dpd beads, we first select the appropriate coarse-graining level, or equivalently, the length scale *r*_c_. Due to the disparate sizes of embs, which can be as large as several micrometers, and gvs, where the diameter is at most several hundred nanometers, we use two different sets of fundamental scales (length, energy, and mass), specified in Table S1 of Supporting Information.

We choose the length scale *r*_c_ so that the radius of the particular object in the smallest dimension is at least 2 *r*_c_. This is to ensure a large enough resolution of the immersed objects compared to the cutoff 1 *r*_*c*_ of the dpd interaction between the beads, eqs S79 and S80 of Supporting Information. The mass scale *m* is chosen to reproduce the density of the water/gas/shell and is calibrated, so that the mass of the water bead is equal to 1 *m*: *m* = ρ_w_^exp^r_c_^3^/ρ_w_, where ρ_w_ is the number density of water beads and ρ_w_^exp^ = 997 kg/m^3^ is the physical density of water. For the energy scale ε, we take the thermal energy at room temperature *T*_0_ = 300 K, ε = *k*_B_ *T*_0_. The time scale τ follows from the other three fundamental scales

6 $$\tau =\sqrt{\frac{mr_{c}^{2}}{\varepsilon }}$$

We use the dpd parameters specified in Table 1. To keep the dpd system fluid-like and avoid the freezing artifacts appearing at *a*_αβ_ ≳ 250 *k*_B_ *T*_0_/*r*_c_,^85–87^ we use *a*_αβ_ = 100 *k*_B_ *T*_0_/*r*_c_. For high coarse-graining levels, as is the case here, such a value of *a*_αβ_ leads to a highly compressible liquid or equivalently a low value of the speed of sound *c*_0_. Since we are interested in phenomena characterized by a low Mach number Ma = *u*/*c*_0_ ≪ 1, with *u* the typical particle velocity, this does not have a great impact on dynamics in this work.

We set the dpd interaction coefficients *a*_wg_, *a*_oo_ and *a*_og_ in line with existing red blood cell models. We should note that setting *a*_wg_ and *a*_og_ to zero results in zero surface tension between the corresponding phases. The surface tension of the interfaces can be tuned by introducing an exponential conservative interaction between the beads of different phases,^88,89^ however this is beyond the scope of the current paper. Additionally *a*_gg_ was set to zero to model the ideal gas with a linear pressure density relation, which we discuss in detail below in subsection “Inducing compression and buckling”.

We have also ensured that the water/gas viscosity ratio is high by varying γ_gg_ and the kernel power *k*_gg_ in eq S80 of Supporting Information. Our choice of dpd parameters yields realistic viscosity ratios η_w_/η_*N*2_ ≈ 63 for water/nitrogen and η_w_/η_air_ ≈ 48 for water/air, which are close to the experimental values of η_w_/η_*N*_2__ ≈ 51 and η_w_/η_air_ ≈ 48.

### 2.4 Down-Scaling of Elastic Forces

At typical length scales *r*_*c*_ of the objects, the dimensionless values of the 2D Young’s moduli are large, as they scale as ∼*r*_c_^2^/ε, i.e., physically, elastic energy is much larger than *k*_B_ *T*_0_. To ensure computational feasibility, we scale down all elastic moduli in our simulations by a factor *f*_scale_ ≪ 1. This preserves the so-called Föppl-von-Karman number FvK, which determines the shape of the objects in equilibrium^90,91^

7 $$FvK=\frac{ER_{0}^{2}}{\kappa }$$

where *E* is the 2D Young’s modulus, κ ∝ *E* is the bending constant, and *R*_0_ is the typical radius of the object. Consequently, computed quantities such as critical stretching forces and buckling pressures must be divided by *f*_scale_.

The behavior of elastic objects under shear flow is governed by the dimensionless capillary number^91,92^

8 $$Ca=\frac{\eta \mathrm{\gamma ̇}R_{eff}}{\mu }$$

To preserve it, the viscosity of water must also be scaled accordingly, ${\mathrm{\eta ̃}}_{w}=f_{scale}\eta_{w}$ , where η_w_ is the physical viscosity of water.

We choose two different *f*_scale_ values corresponding to each unit set (Table S1), ensuring that κ/(*k*_B_ *T*_0_) > 10 to prevent significant perturbation of the object by thermal fluctuations, which primarily originate from the solvent beads. For each unit set, the target viscosity η̃ is achieved by adjusting the values of γ_ww_ and *k*_ww_ (Table 1).

### 2.5 Fluid–Structure Interactions

The boundary conditions at the fluid-immersed structure interface have a large effect on the behavior of ucas under nonequilibrium conditions, such as shear or plug flow.^93^ First, to prevent leakage of the solvent inside the gas vesicles or microbubble, we impose the no-through boundary condition by using the bounce-back mechanism, where the particles are introduced back based on the Maxwell distribution of velocities at temperature *T*_0_.^94^

To control the velocity boundary conditions at the uca–water or uca–gas interface, one typically tunes the dissipative parameter γ_ow_ (γ_og_) between the water (gas) and the uca beads. A specific value of the dissipative parameter ensures the no-slip boundary condition^48^

9 $$\gamma_{o\left\{w,g\right\}}=\frac{2{\mathrm{\eta ̃}}_{\left\{w,g\right\}}\left(2k_{fsi}+1\right)\left(2k_{fsi}+2\right)\left(2k_{fsi}+3\right)\left(2k_{fsi}+4\right)}{3\pi r_{c}^{4}\rho_{\left\{w,g\right\}}\rho_{m}}$$

where ρ_m_ is the area number density of the vertices of the immersed membrane, ρ_{w,g}_ is the number density of the fluid (water or gas), ${\mathrm{\eta ̃}}_{\left\{w,g\right\}}$ is the corresponding scaled-down viscosity, and *k*_fsi_ is the kernel power of the dissipative weight function set to 0.5 for both unit sets. The complete dpd parameter sets used for uca–water and uca–gas interfaces are given in Table 1.

### 2.6 Inducing Compression and Buckling

According to the semiempirical equation of state eq 5, the fluid pressure is linearly proportional to *a*_ww_, which is confirmed numerically (see Figure S9 in Supporting Information). Thus, water pressure is controlled by varying the interaction parameter *a*_ww_ between water beads linearly in time until it reaches a desired final value, where it remains for the second half of the simulation to allow the structure to relax into its final shape.

Unlike that of water, the compressibility of the gas phase plays an important role, significantly affecting the shell’s collapse pressure. At the buckling transition, the dominant deformation mode is determined by the bending constant (which is small for thin shells) and is thus sensitive to the compressibility of the interior gas. At realistic pressure, the compressibility of dpd gas, as follows from the dpd equation of state eq 5, is anomalously low. To remedy this, we model the gas phase as ideal by setting the interactions between gas beads to 0, which also aligns with the ideal gas behavior in the relevant temperature and pressure range. Consequently, the resulting dpd gas pressure is extremely low. To ensure mechanical stability, it is compensated by applying an outward force to each triangular face of the shell, evenly distributed among its three vertices to ensure zero torque on the triangle: **f**_p_ = −*pA* **n**/3, where *A* and **n** are the area and inward-pointing normal of the triangle (see Supporting Information S2), and *p* is the desired pressure compensation.

### 2.7 Open-Boundary Molecular Dynamics

To perform nonequilibrium simulations we use open-boundary molecular dynamics (obmd),^95–97^ which allows imposing momentum and/or heat fluxes at the system’s boundaries. A typical obmd setup consists of three fundamental parts: a central domain—the region of interest (roi), and two buffer regions in which particle deletion or insertion is performed. The particle number in the buffer regions is controlled by a feedback algorithm

10 $$\Delta N=-\frac{\Delta t}{\tau_{B}}\left(N-\alpha_{B}N_{0}\right)$$

where Δ*N* is the number of particles to be deleted (Δ*N* < 0) or inserted (Δ*N* > 0), Δ*t* and τ_B_ are the time step and relaxation time of the buffers, *N* and *N*_0_ the current and equilibrium particle numbers in either buffer, and α_B_ an empirical customized parameter. Particle insertion is facilitated by the usher algorithm,^98^ which employs an iterative steepest descent scheme on the potential energy surface.

obmd imposes boundary conditions by adding external forces **f**_*i*_ to all buffer particles *i*, determined from the momentum balance

11 $$JA\cdot n=\sum_{i}f_{i}+\sum_{i^{\prime }}\frac{\Delta \left(m_{i^{\prime }}v_{i^{\prime }}\right)}{\Delta t}$$

where **J** is the momentum current density tensor, *A* the surface area of the interface between the buffer and the roi, and **n** the normal to this interface pointing toward the center of the roi. The second sum in eq 11 stands for the momentum gain or loss upon insertion or deletion of particles *i*′ in the current time step. The momentum flux across the buffer–roi interface is dictated by the desired boundary condition for the stresses, e.g. on boundaries with normals **n** = ± ${ê}_{x}$

12 $$J\cdot n=P_{xx}n+P_{yx}{ê}_{y}+P_{zx}{ê}_{z}$$

where *P*_*xx*_ is the equilibrium pressure, and *P*_*yx*_, *P*_*zx*_ the shear stress components.

### 2.8 Quasiharmonic Analysis

In the analysis of molecular dynamics simulations of biomolecules, extracting eigenmodes and their frequencies is essential. The well-established method for this is quasiharmonic analysis,^99–101^ also sometimes referred to as principal component analysis (pca).^102^ This approach is not limited to molecules but can also be applied to coarse-grained models of elastic objects, such as carbon nanotubes,^103,104^ and in our case to ucas.

The thermalized equilibrium configurational probability distribution of membrane vertices is given by

13 $$P\left(x\right)=\frac{e^{-\beta E\left(x\right)}}{\int e^{-\beta E\left(x\right)}\;dx}$$

where *E*(**x**) is the potential energy of the configuration expressed by a supervector **x** of particle coordinates, and β = 1/(*k*_B_ *T*_0_). In the quasiharmonic approximation, the potential energy surface is assumed to be a quadratic function $E\left(x\right)\approx E\left(\langle x\rangle \right)+\frac{1}{2}{\left(x-\left\langle x\right\rangle \right)}^{T}V\left(x-\left\langle x\right\rangle \right)$ , where $V_{ij}=\frac{\partial^{2}E}{\partial x_{i}\partial x_{j}}$ is the Hessian, and ⟨.⟩ denotes thermal average, which is in our case calculated as a time-average of the vertex positions. Within this approximation, the configurational probability function takes the form of a multivariate Gaussian distribution and therefore the Hessian can be extracted from the trajectory through the covariance matrix Σ_*ij*_ = ⟨(*x*_*i*_ – ⟨*x*_*i*_⟩)­(*x*_*j*_ − ⟨*x*_*j*_⟩)⟩ of particle coordinates

14 $$V=k_{B}T_{0}\Sigma^{-1}$$

In determining Σ, we subtract the motion of the center of mass of the object and remove rotational motion using a trajectory alignment algorithm from the MDAnalysis Python package.^105,106^ This algorithm finds the optimal rotation matrix between the current and reference configurations by minimizing the root-mean-square deviation between the configurations.^107,108^

Ignoring the damping coming from the solvent and the gas, the equation of motion reads

15 $$M\overset{..}{x}+Vx=0$$

where **M** is the diagonal mass matrix, **M** = *m* **I** in our case. Using the ansatz **x** = **x**_0_ *e* ^iω*t*^, one obtains the generalized eigenvalue problem

16 $$\left(\Sigma -\frac{k_{B}T_{0}}{\omega^{2}}M^{-1}\right)x_{0}=0$$

which can be recast into a standard eigenvalue problem by defining $q_{0}=\sqrt{M}x_{0}$ and $\Sigma^{\prime }=\sqrt{M}\Sigma \sqrt{M}$

17 $$\left(\Sigma^{\prime }-\lambda^{\prime }I\right)q_{0}=0$$

where the eigenfrequencies of the structural vibrations

18 $$\omega =\sqrt{\frac{k_{B}T_{0}}{\lambda^{\prime }}}$$

are obtained from the eigenvalues λ′.

## 3 Results and Discussion

### 3.1 General Particle-Based Elasticity Framework for Simulating Membrane-Encapsulated Soft- and Biomaterials

The role of membranes in soft- and biomaterials is multifaceted. For biological cells, the membrane separates the interior from external disturbances and can also provide means for the exchange of ions, solvents, gas molecules, and other substances.^109^ Biological membranes typically consist of lipid, polymer, or protein units. The type of these units, their interaction with each other and the environment, as well as their bonding topology dictate the elastic behavior of membranes.

This extreme diversity of membranes with different elastic properties requires a general methodology that is capable of incorporating various possible symmetries, for example, the isotropic elasticity of embs on one hand, and orthotropic elasticity of gvs on the other. There are different approaches to modeling elastic properties of thin shells. A popular one is connecting the various subunits with harmonic bonds or potential wells, aided by a harmonic angular potential.^110^ It is well-known that a straightforward application of such an approach—using harmonic bonds with equal spring constants—does not lead to general elastic behavior.^111^ An alternative approach, which we follow here, is to discretize the continuum elastic surface energy on a triangulated surface spanned by the various subunits (see Figure 1).^112^ This energy expression is then used in the subsequent per-vertex force calculations.

**Figure 1:**
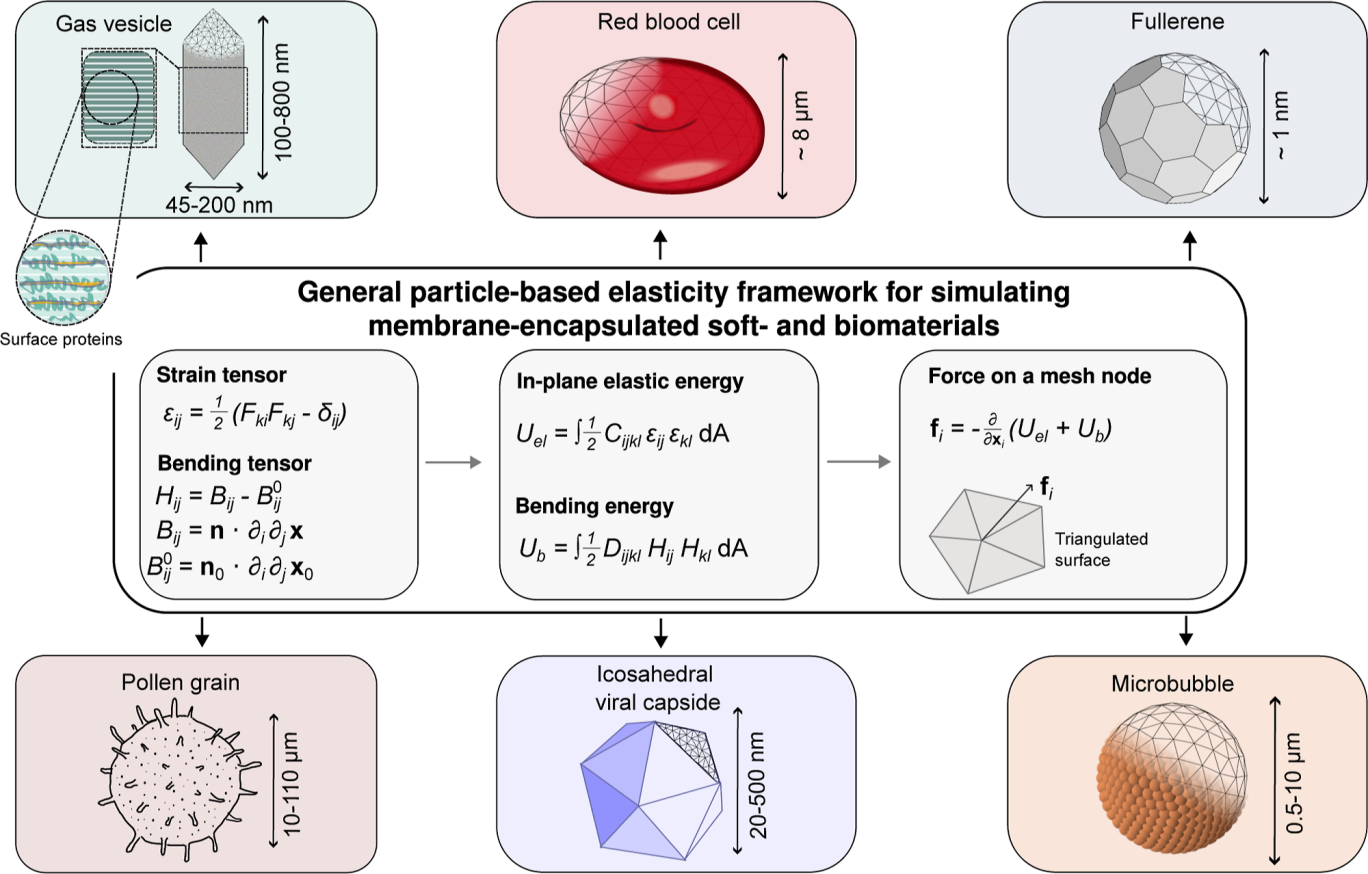
Overview of particle-based elastic modeling of thin shells used in this work. An object is described by its subunits and a triangulated network. Elastic energy is split into two parts: in-plane elastic energy, characterized by the material elastic tensor *C*_*ijkl*_ and the strain tensor ε_*ij*_ measuring the deformations; and bending energy, characterized by the flexural rigidity tensor *D*_*ijkl*_ and the bending tensor *H*_*ij*_. The corresponding vertex forces are calculated by taking derivatives of the total elastic energy with respect to vertex positions. The modeling framework is applicable to biomaterials of different shapes and general local anisotropy.

We quantify the deformation using the deformation gradient $F_{ij}=\frac{\partial x_{i}}{\partial x_{0j}}$ , which relates the difference in positions of two infinitesimally close material points in the deformed configuration **x** to their difference in the reference configuration **x**_0_.^113^ To exclude local rotation, deformation is typically described using the Green-Lagrange deformation tensor (strain tensor) $\varepsilon =\frac{1}{2}\left(F^{T}F-I\right)$ , which measures the deformation relative to the reference configuration. Moreover, we incorporate anisotropic elasticity to model the diverse and generally complex elastic properties of biological membranes. Anisotropic objects are characterized by their reduced symmetry group or equivalently by a set of structural tensors **M**, which reflect the distinguished directions, lines, or planes of an object. See Supporting Information for a detailed overview of the effects of material symmetry and the principle of isotropy of space on the form of the elastic energy.

In linear elastic theory of thin shells, in-plane and bending deformations are decoupled, and the total elastic energy is *U* = *U*_el_ + *U*_b_, where *U*_el_ and *U*_b_ are the in-plane elastic and bending energies, respectively.

The general in-plane elastic energy is formulated using the two-dimensional (2D) in-plane strain tensor **ε**

19 $$U_{el}=\int \frac{1}{2}C_{ijkl}\varepsilon_{ij}\varepsilon_{kl}dA$$

where d*A* is the surface element of the shell, and *C*_*ijkl*_ = *hC*_*ijkl*_^3D^ is its in-plane elastic tensor, derived from the three-dimensional (3D) material elastic tensor *C*_*ijkl*_^3*D*^ and the shell thickness *h*, eq S6 in Supporting Information. The in-plane stress tensor σ_*ij*_ = *C*_*ijkl*_ε_*kl*_ has units of force per unit length. The tensor *C*_*ijkl*_ satisfies *C*_*ijkl*_ = *C*_*jikl*_ = *C*_*ijlk*_, which reflects the symmetries of the strain and stress tensors. Under the assumption of hyperelasticity, where stress is derived from an elastic potential, it also satisfies *C*_*ijkl*_ = *C*_*klij*_. These symmetries reduce the maximum possible number of elastic parameters from 81 to 21 in 3D and from 16 to 6 in 2D. The in-plane elastic tensor *C*_*ijkl*_ is expressed as a combination of Kronecker delta δ_*ij*_ and in-plane structural tensor(s) *M*_*ij*_, as derived in Supporting Information in the context of orthotropic elasticity of gvs.

A general form of the bending energy is

20 $$U_{b}=\int \frac{1}{2}D_{ijkl}H_{ij}H_{kl}dA$$

where *H*_*ij*_ = *B*_*ij*_ – *B*_*ij*_^0^ is the bending tensor,^114^ which measures the deviation of the curvature tensor *B*_*ij*_ = **n** · ∂_*i*_∂_*j*_ **x** in the deformed state from the spontaneous curvature tensor *B*_*ij*_^0^ = **n**_0_ · ∂_*i*_∂_*j*_ **x**_0_. Here, **n** and **n**_0_ denote the normals to the deformed (**x**) and undeformed (**x**_0_) configuration surfaces, respectively. The principal directions of the curvature tensor align with extremal curvatures, represented by its eigenvalues 1/*R*_1_ and 1/*R*_2_, where *R*_1_ and *R*_2_ are the principal radii of curvature. In linear thin shell elasticity, the material flexural rigidity tensor *D*_*ijkl*_ is fully specified by the material elastic tensor through the relation $D_{ijkl}=\frac{h^{3}}{12}C_{ijkl}^{3D}=\frac{h^{2}}{12}C_{ijkl}$ , eq S7 in Supporting Information.

In the constant strain triangle approximation (cst),^115^ where the strain field ε is assumed to be constant within each triangle, the surface integrals eqs 19 and 20 can be replaced by summation over the triangles of the triangulated surface, as given in eqs S8 and S33 of Supporting Information.

### 3.2 Models of Ultrasound Contrast Agents

Within the introduced general elastic particle-based computational framework, we focus on modeling the behavior of two encapsulated agents: embs and gvs. The specific models we employ are inspired by rbc membrane models.^40,41,43,44,47,49–51,54,55,91,116–118^ The rbc membrane consists of two main components: a pseudohexagonal elastic spectrin network,^119^ and a fluid-like lipid bilayer. In contrast, polymer- or protein-based embs and gvs comprise only an elastic network,^29^ while lipid-based microbubbles are encapsulated by a lipid monolayer membrane.^72^ The primary differences in modeling embs and gvs compared to rbcs lie in the different topology of the triangulated surfaces and, in the case of gvs, the inclusion of anisotropic elastic terms.

### 3.3 Microbubbles

emb shells are made of proteins, polymers, or lipids. Most embs appear to be well described by an isotropic elastic model,^120^ although there are continuum models that assume transversely isotropic elastic shells where the anisotropy axis is along the radial direction.^121^

The elastic tensor *C*_*ijkl*_ of isotropic materials is exclusively expressed through the isotropic tensor—the Kronecker delta δ_*ij*_. It has two independent terms

21 $$C_{ijkl}=K_{a}\delta_{ij}\delta_{kl}+\mu \left(\delta_{ik}\delta_{jl}+\delta_{il}\delta_{jk}-\delta_{ij}\delta_{kl}\right)$$

where *K*_a_ and μ are the bulk and the shear moduli, respectively. There is a more detailed description of the elastic moduli and the expression for elastic energy in Supporting Information.

The bending energy follows from eq 20 and, for isotropic shells, consists of two independent terms

22 $$U_{b}=\int \left[\frac{1}{2}\kappa J_{1}^{2}-\kappa \left(1-\nu \right)J_{2}\right]dA$$

where $\kappa =\frac{Eh^{2}}{12\left(1-\nu^{2}\right)}$ is the bending constant, *E*, ν are the 2D Young’s modulus and Poisson’s ratio (eqs S20 and S21 in Supporting Information), and the scalar differential curvature invariants are defined as *J*_1_ = tr­(**H**) and $J_{2}=\frac{1}{2}\left[tr{\left(H\right)}^{2}-tr\left(H^{2}\right)\right]=\det\left(H\right)$ . The bending energy eq 22 is discretized using the Kantor-Nelson approach, see Supporting Information.

### 3.4 Gas Vesicles

The gv membrane consists of GvpA protein ribs arranged helically around the gv axis,^34,36^ see Figure 1. Its elastic properties differ along the ribs and perpendicular to them.^34,65^ The local material symmetry group G is therefore orthotropic, spanned by four elements: identity, inversion and a pair of reflections across the rib direction and perpendicular to it. The 2D elastic tensor can therefore be constructed using Kronecker delta δ_*ij*_ and one structural tensor, which is invariant to all group elements in G : **M** = **m** ⊗ **m**, where **m** is chosen to point perpendicular to the rib. Since the ribs run nearly perpendicular to the gv axis, **m** is well approximated by a projection (see Supporting Information).

Taking into account this symmetry, the most general form of the elastic tensor is

23 $$\begin{array}{ll} C_{ijkl} & =K_{a}\delta_{ij}\delta_{kl}+\mu \left(\delta_{ik}\delta_{jl}+\delta_{il}\delta_{jk}-\delta_{ij}\delta_{kl}\right)\\ & +\left(\mu_{L}-\mu \right)\left(m_{i}m_{l}\delta_{jk}+m_{j}m_{l}\delta_{ik}+m_{i}m_{k}\delta_{jl}+m_{j}m_{k}\delta_{il}\right)\\ & +cm_{i}m_{j}m_{k}m_{l}, \end{array}$$

with μ_L_ > 0, *c* > 0 ^122,123^ the anisotropic elastic coefficients, which are positive for stability reasons. The coefficient μ_L_ is the membrane’s (in-plane) shear elastic constant, while *c* contributes to the stiffness along the anisotropy axis **m**.^124^ A more in-depth explanation regarding the in-plane elastic energy is provided in Supporting Information. Our construction of the elastic tensor assumes isolated, fully formed shells with a fixed equilibrium helical arrangement of GvpA proteins. Modeling of the elastic properties of gvs during the biogenesis or under variable temperature conditions, where the shell is forming through dynamic self-assembly of GvpA proteins, is beyond the scope of this paper.

In the linear regime, the coefficients *K*_a_, μ, μ_L_, and *c* in eq 23 can be related to the engineering constants: Young’s modulus along the gv axis, *E*_l_, and perpendicular to it (along the ribs), *E*_t_, Poisson’s ratio ν_lt_ for stretching along the ribs, and shear modulus *G*(=μ_L_). The other Poisson’s ratio ν_tl_ is already fixed with these choices. These relations are given in eqs S24–S31 of S1.

Following eq 20, the bending energy of a thin orthotropic shell can be expressed as a sum of four independent terms

24 $$U_{b}=\int \left(\frac{1}{2}\kappa_{t}J_{1}^{2}-\kappa_{t}\left(1-\nu_{lt}\right)J_{2}+\kappa_{\mu }J_{3}+\frac{1}{2}\kappa_{c}J_{4}^{2}\right)dA$$

where new scalar differential curvature invariants are defined as *J*_3_ = **m** ^*T*^ **H** ^*T*^ **Hm**, *J*_4_ = **m** ^*T*^ **Hm**, and $\kappa_{t}=\frac{E_{t}h^{2}}{12\left(1-\nu_{lt}\nu_{tl}\right)}$ , $\kappa_{\mu }=\frac{\left(\mu -\mu_{L}\right)h^{2}}{6},$ $\kappa_{c}=\frac{ch^{2}}{12}$ are the bending constants in the thin shell regime.^125^

For simplicity, we keep the bending energy of the gv membrane isotropic in this work and set the bending constant to $\kappa =\frac{E_{t}h^{2}}{12\left(1-\nu_{lt}^{2}\right)}$ , where we have used the smaller Young’s modulus *E*_t_. This is to ensure that the circumferential instability, which in buckling experiments occurs at lower pressure amplitudes than other more complicated instabilities, has the correct energy cost.

### 3.5 Mechanical Properties

Mechanical properties of biomaterials can be determined by performing elementary mechanical experiments, such as stretching,^126^ torsion,^127^ and compression.^128^ Stretching experiments are typically done using optical tweezers, where two micrometer-sized spherical silica beads are attached to the ends of a particle and then moved in opposite directions. The experimental data consist of force–displacement curves, which can be translated to stress–strain curves under certain assumptions.

We conducted stretching, compression/buckling, and torsion simulations of embs and gvs. The stretching force–displacement curves of our model embs and gvs were determined by adding oppositely equal forces to a small set of diametrically opposite vertices of their membrane. The gv torsion simulations were performed by rotating the gv cylinder ends in opposite directions, Figure S7. In principle, this could be achieved experimentally with an angular optical tweezer device, which is a relatively new methodology.^129,130^

We also derive analytical results for the stretching, torsion, and compression in the small deformation limit and verify their validity by simulations.

### 3.6 Microbubble Stretching

We model the stretching experiment by uniformly distributing the stretching forces over 64 vertices at each pole of an emb (approximately 2.5% of all the vertices). This is equivalent to a contact diameter of approximately 0.65 μm, which falls within a range of typical silica bead sizes used in optical tweezer experiments. As shown in Figure 2a, the stretching of an emb results in a decrease in diameter *D* perpendicular to the stretching direction. For small strains, one finds (eq S65 of Supporting Information)

25 $$D=D_{0}-\frac{F_{tot}}{2\pi \mu }$$

where *F*_tot_ is the total force applied to the ends of the emb, and *D*_0_ = 2*R*_0_ is its equilibrium diameter. As seen in Figure 2a,b, the measured diameter perpendicular to the stretching direction matches excellently with eq 25 in the regime of small strains. The expression eq 25 differs from the result for an elastic disc, which is often used to model red blood cell stretching in the linear regime:^131^ $D=D_{0}-\frac{F_{tot}}{2\pi \mu }\left[1-\left(1-\frac{\pi }{2}\right)\frac{\mu }{K_{a}}\right]$ .

**Figure 2:**
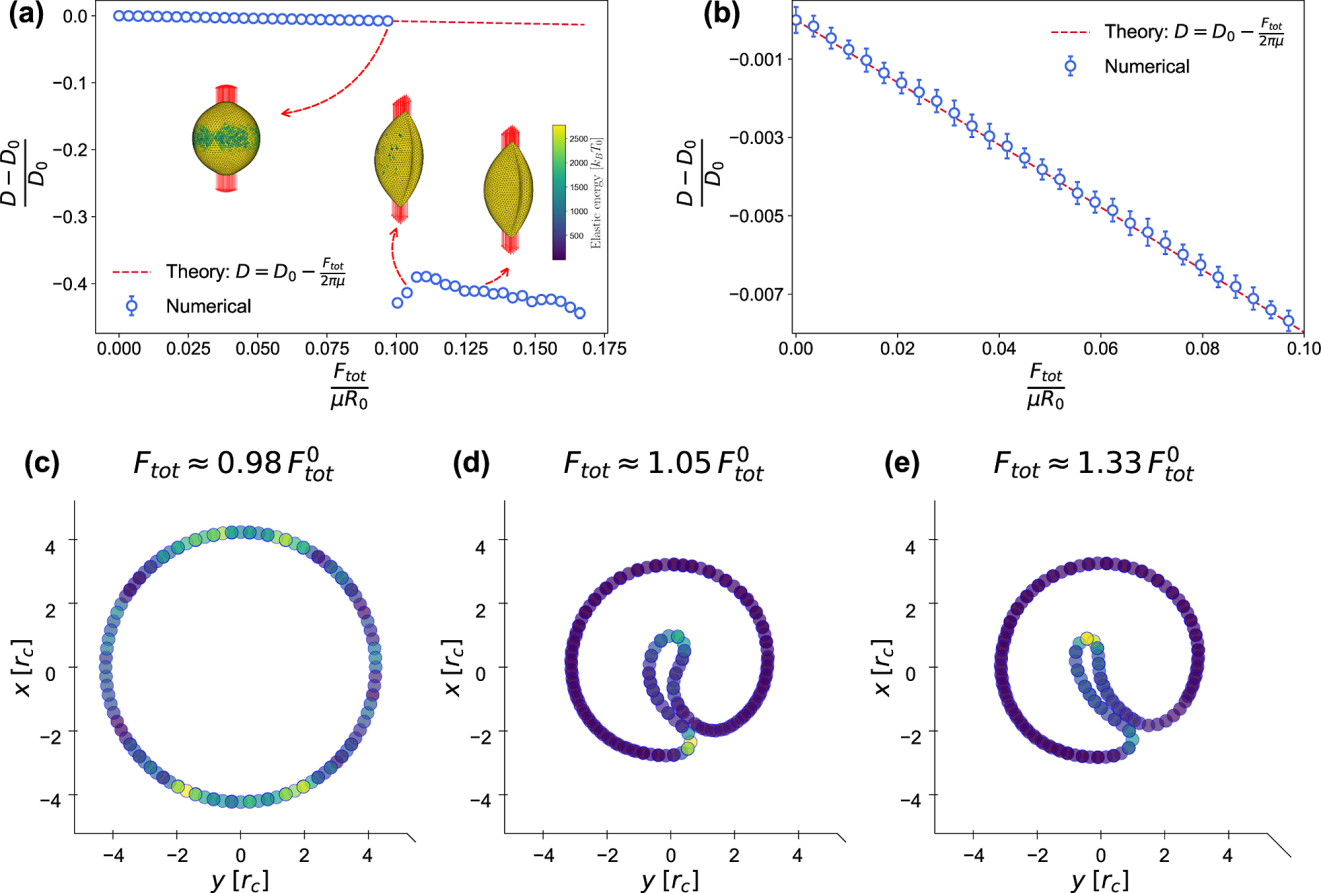
emb stretching. (a) Relative emb diameter change as a function of applied force *F*_tot_ in units of μ*R*_0_. (b) Small-strain part of the dependence in (a), compared to eq 25. (c–e) Equatorial slices of embs perpendicular to the stretching direction at values of *F*_tot_, corresponding to the three regimes in (a); *F*_tot_^0^ = 0.099 μ*R*_0_. The coloring in the side views (a) the top views (c–e) represents local elastic energy. Equatorial slices (c–e) are single-particle thick.

Above *F*_tot_ ≈ 0.099 μ*R*_0_, the emb undergoes a transition into a circumferentially wrinkled shape and the diameter, calculated as twice the average distance of equatorial particles from their center of mass, drops significantly.

The onset of this wrinkling transition can be understood as a trade-off between compression in the circumferential direction and out-of-plane bending deformation. When stretching is applied, the shell initially accommodates the deformation through in-plane stretching and accompanying in-plane compression in the equatorial direction. Eventually, this compression becomes too costly and destabilizes. At this point the shell reduces the compression energy by buckling.

One can also notice an azimuthally nonuniform distribution of energy of the equatorial slice in Figure 2c. These variations appear random and reflect the fluctuations in the membrane as it approaches the wrinkling transition.

### 3.7 Gas Vesicle Stretching

We examine the response of a gv to stretching along its axis (*z* axis), Figure 3, which should be the most feasible experimentally. In these simulations we distribute the stretching forces uniformly over all vertices of the conical parts. For small stretching forces, the length of the gv increases linearly, while its diameter in the *xy* plane decreases, in line with simple linear elastic response. The stress–strain response of a gv can be estimated by considering the uniaxial stress in its cylindrical part, $\sigma_{zz}=\frac{F_{tot}}{2\pi R}$ , and the corresponding strain along *z*, ε_*zz*_=(*H*_cyl_ – *H*_cyl_^0^)/*H*_cyl_^0^, where *H*_cyl_ and *H*_cyl_^0^ are the heights of the gv cylinder in the deformed and reference configurations, respectively. The circumferential strain, on the other hand, is deduced from the relative radius change, ε_φφ_ = (*R* – *R*_0_)/*R*_0_, where *R* and *R*_0_ are the radii in the deformed and reference configurations, respectively. It is important to emphasize that *H*_cyl_ is measured between the ends of the cylindrical section, excluding the conical parts, where the stress depends on *z* as the radius tapers from *R* to 0. Figure 3a,b shows that the linear response matches excellently with the constitutive equations eqs S74 and S75 of Supporting Information. Measurement of this small-strain response could therefore be used to estimate both the longitudinal Young’s modulus *E*_l_ and the Poisson’s ratio ν_lt_.

**Figure 3:**
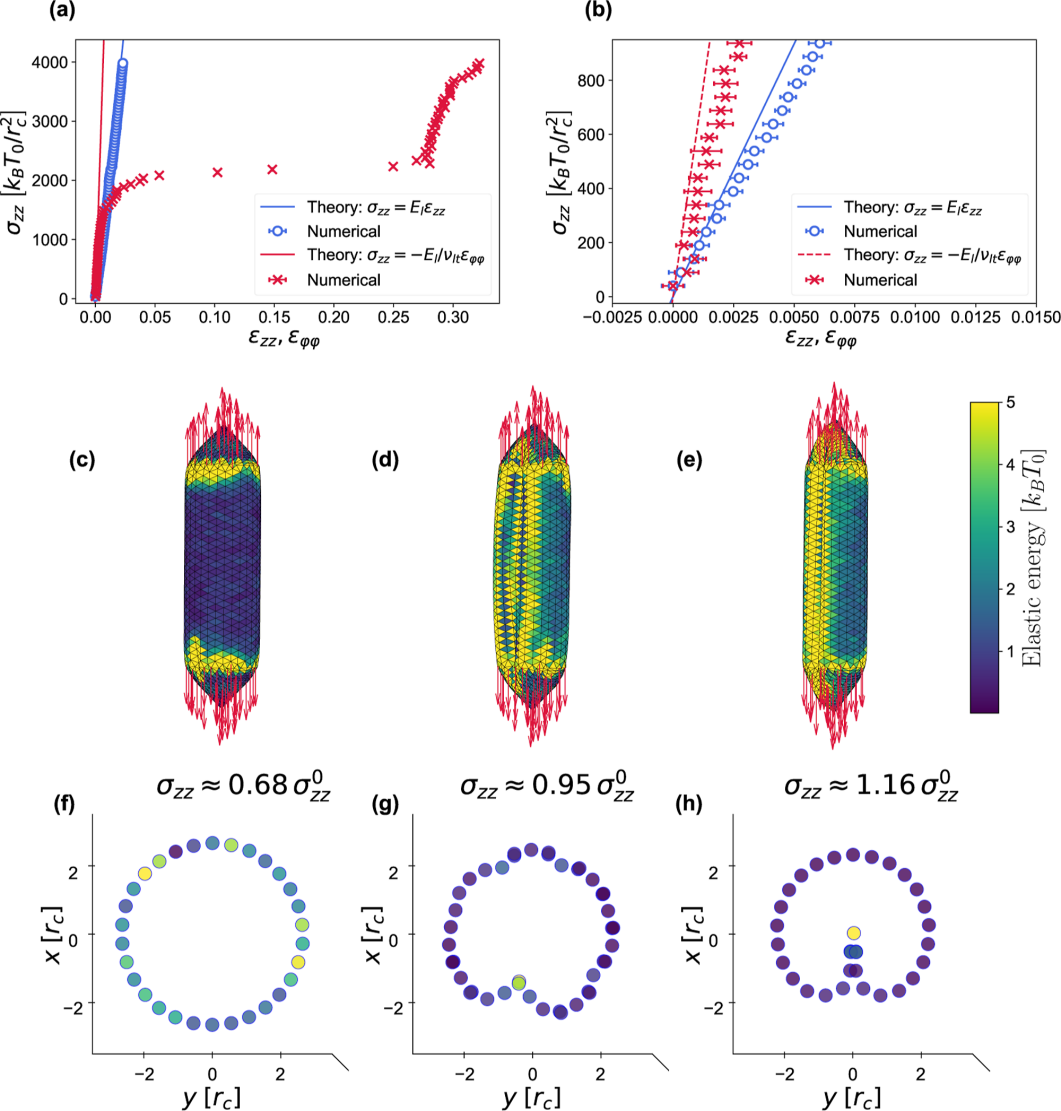
gv stretching. (a) Stress σ_*zz*_ as a function of longitudinal strain ε_*zz*_ and circumferential strain ε_φφ_ for a gv stretched along its axis. (b) Comparison with eqs S74 and S75 of Supporting Information in the limit of low strain. (c–e) Side views and (f–h) central *xy* slices of the gv at different values of σ_*zz*_, with σ_*zz*_^0^ = 2184 *k*_B_ *T*_0_/*r*_*c*_^2^. The coloring represent local elastic energy. To better depict the first buckling transition, slice (g) is taken at a height of 4 *r*_c_ from the gv center. Central slices (f–h) are single-particle thick.

At a certain point, the gv buckles, forming a dimple, localized to each end of the cylindrical part, see Figure 3d,g. The onset of these dimples is already seen in Figure 3c which shows an azimuthally nonuniform distribution of energy at the ends of the cylindrical parts. At a larger longitudinal stress $\sigma_{zz}^{0}\approx 2184\,\;k_{B}T_{0}/r_{c}^{2}$ , the gv buckles into a shape with a single dimple along the entire length, Figure 3e,h. The onset of this instability is analogous to that in the stretching of embs—the circumferential compression of the gv reaches a point, where out-of-plane bending is energetically favorable. This transition is also accompanied by a large jump in the circumferential strain ε_φφ_. Interestingly, the longitudinal strain ε_*zz*_ keeps increasing linearly with σ_*zz*_ without a large discontinuity.

### 3.8 Compression

In compression experiments, the external fluid pressure is increased and the corresponding volume change of the body is measured. In simulations, the pressure increase Δ*p* is controlled by varying the interaction parameter *a*_ww_ (eq 1) between the water beads.

For low Δ*p*, the volume change can be obtained analytically for both embs and gvs by calculating the stresses σ_θθ_ and σ_φφ_, depicted in Figures 4 and 5. Neglecting the compressibility modulus of the trapped gas and the membrane bending contributions, which are negligible in the regime of low deformation, the volume strain of an emb is (eqs S66–S71 of Supporting Information)

26 $$\frac{\Delta V}{V_{0}}=-\frac{3\left(1-\nu \right)R_{0}}{2E}\Delta p=-\frac{3R_{0}}{4K_{a}}\Delta p$$

which is confirmed numerically in Figure 6a,b.

**Figure 4:**
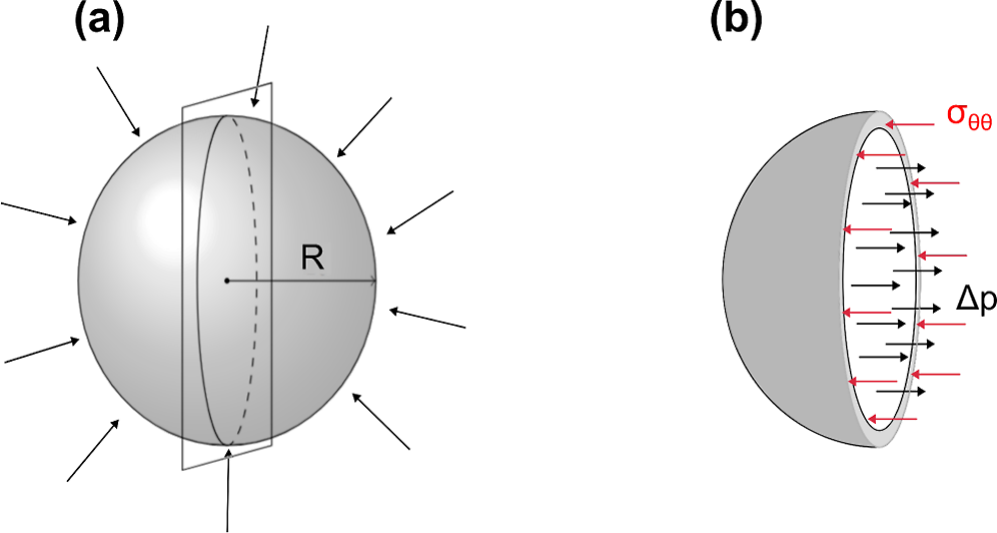
Pressure-induced stress in the emb membrane. (a) emb in a pressurized environment, exerting uniform compression forces. (b) Differential pressure force on the hemisphere (black arrows) in equilibrium with elastic force of the shell (red arrows).

**Figure 5:**
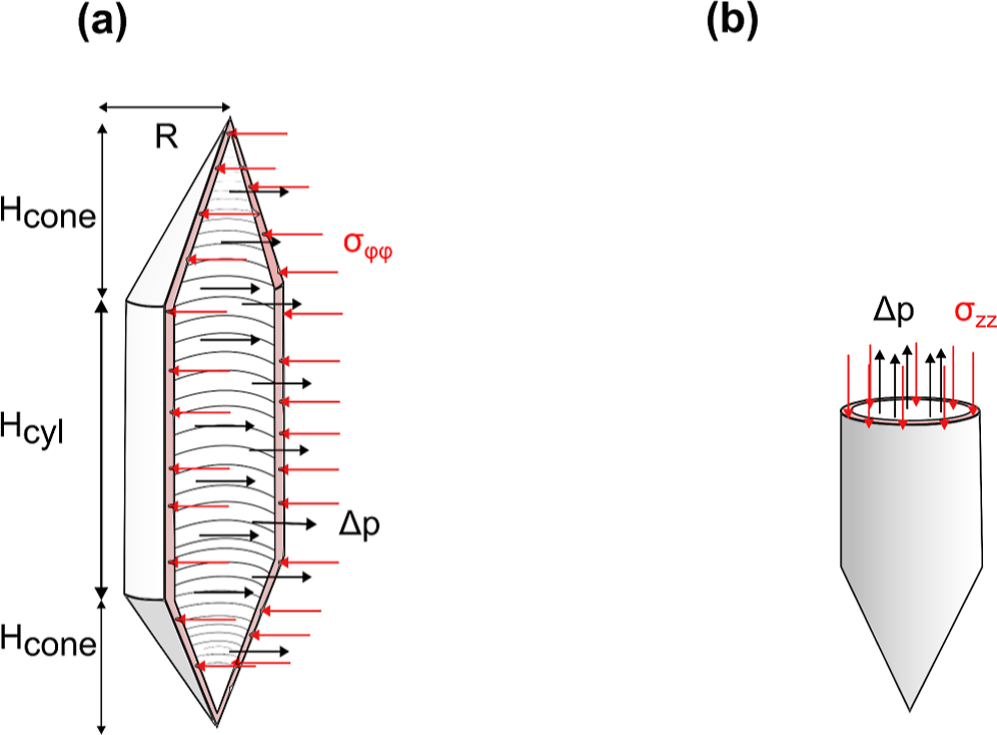
Pressure-induced stress in the gv membrane. gv in a pressurized environment: (a) meridional stress σ_φφ_ caused by differential pressure on the longitudinal cross-section, and (b) equatorial stress σ_*zz*_ caused by differential pressure on the transverse cross-section.

**Figure 6:**
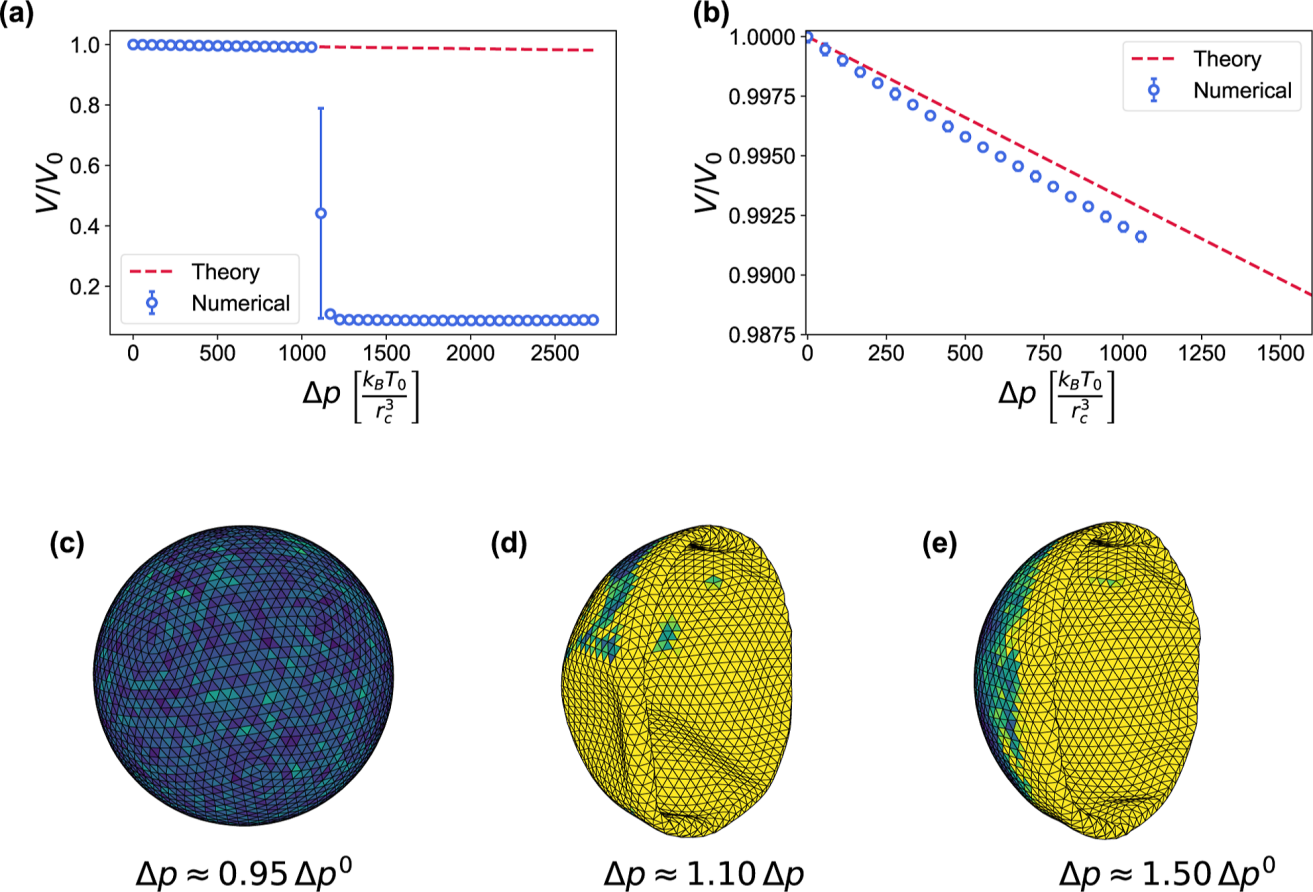
emb compression and buckling. (a) Relative volume change of an emb due to the external pressure increase Δ*p*, compared to eq 26 (red dashed line). (b) Closeup of (a) in the small deformation regime. (c–e) Compressed emb at different values of Δ*p*, with Δ*p* ^0^ = 1113 *k*_B_ *T*_0_/*r*_c_^3^ the buckling threshold pressure. Local buckling nucleations are noticeable in (c), while (d,e) show fully buckled states. The coloring represents local elastic energy.

For a gv with orthotropic elasticity, the volume strain depends on both Young’s moduli *E*_l_ and *E*_t_, and the Poisson’s ratio ν_*lt*_ (eqs S76–S78 of Supporting Information)

27 $$\frac{\Delta V}{V_{0}}=-\frac{R_{0}}{2E_{l}}\left(1-4\nu_{lt}+4\frac{E_{l}}{E_{t}}\right)\Delta p$$

which is again confirmed numerically in Figure 7a,b. The linear weak-compression slope was also observed experimentally by Walsby^132^ using a glass compression tube and was used to estimate the bulk modulus of gvs. In the isotropic case, where *E*_l_ = *E*_t_, eq 27 agrees with the well-known result for isotropic cylindrical shells,^133^ which is often used to estimate the Young’s modulus of gvs.^29,132^

**Figure 7:**
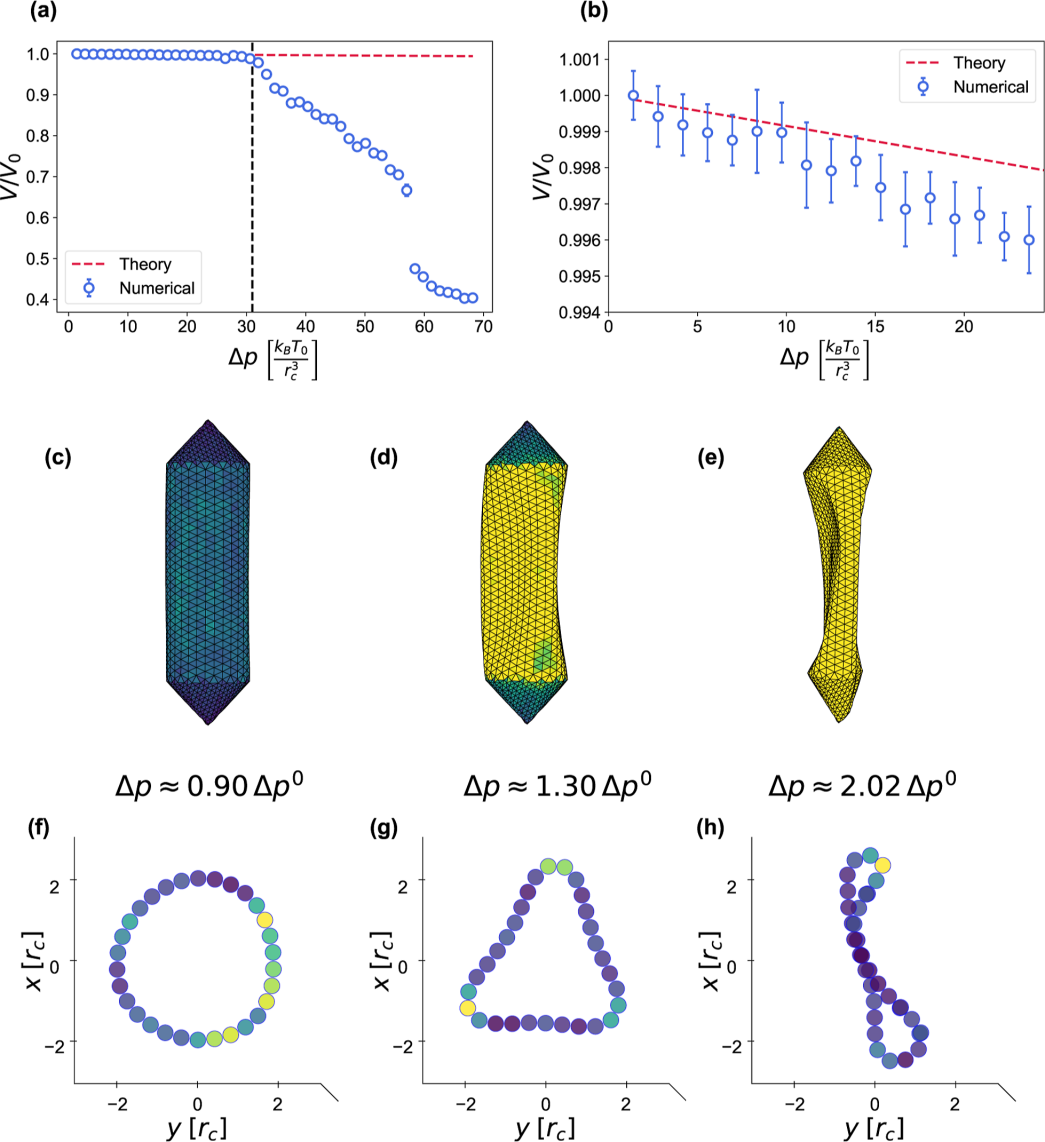
gv compression and buckling. (a) Relative volume change of a gv due to the external pressure increase Δ*p*, compared to eq 27 (red dashed line). The buckling pressure Δ*p* ^0^ = 31.0 *k*_B_ *T*_0_/*r*_c_^3^ is indicated by a black dashed vertical line. (b) Closeup of (a) in the small deformation regime. (c–e) Side views and (f–h) central transverse slices of the gv at different Δ*p*. The coloring represents local elastic energy.

In the small deformation regime governed by the membrane in-plane elasticity, as described by eqs 26 and 27, the effective compressibility of the shell increases linearly with its size *R*_0_. The shape of the shell also plays an important role. There is a distinct difference between the compression of the spherical emb and the cylindrical gv with the same radius. For the same shell material, i.e., *E*_l_ = *E*_t_ = *E*, ν_lt_ = ν, eq 27 gives $\frac{\Delta V}{V_{0}}=-\frac{\left(5-4\nu \right)R_{0}}{2E}\Delta p$ , which should be compared with eq 26. Thus, for the same shell material and radius, the cylinder has almost twice the compressibility of the sphere.

### 3.9 Buckling

gvs are known to produce nonlinear acoustic signals when exposed to high-amplitude ultrasound pulses^20^—at such pressure amplitudes, they buckle reversibly. By measuring optical density as a function of pressure, the buckling pressures were experimentally determined for a wide range of gv sizes and diameters. We investigate buckling instabilities of embs and gvs by increasing the solvent pressure beyond the small strain limit.

Examples of emb buckling are shown in Figure 6c–e. An analytical expression for the critical buckling pressure of spherical shells exists, eq S73 in Supporting Information, which is in an excellent agreement with our simulations, see Figure S6 in Supporting Information. It involves an empirical correction factor due to imperfections of the shell, which consistently takes on a well-defined value also for our model emb. We also performed dynamic numerical buckling experiments at various pressure increase rates. While the buckling pressure remains nearly unchanged across different rates, the structure evolves through different intermediate shapes, from a single indentation for the slowest pressure increase to multiple lobes when the pressure increases more abruptly, which is in line with the numerical simulations of ref 134.

For gvs, we compare the predicted buckling pressure to the hydrostatic critical pressure in ref 66, which considers isolated gvs in a solution. At a pressure of about Δ*p* ^0^ ≈ 31.0 *k*_B_ *T*_0_/*r*_c_^3^, our gv buckles into a shape with three lobes along the circumference, Figure 7d,g. Reverting to physical units and up-scaling by *f*_scale_ (see Methods and Table S1), Δ*p* ^0^ ≈ 37.9 kPa. This is below the value of 64 kPa in ref 66. It is also significantly below experimental values of refs 19 and 64 of about Δ*p* ^0^ ≈ 200 kPa and Δ*p* ^0^ ≈ 178 kPa, respectively. One possible reason for the discrepancies with refs 19 and 64 is that these experiments used acoustic waves to induce the gv collapse, which is known to yield higher measured collapse pressures than hydrostatic techniques. A general reason is that the actual bending constant κ is an independent parameter, lower than predicted by thin-shell theory—a single molecular layer membrane can hardly be considered a continuum across its thickness. Another possible reason is that the nonlinear elastic coefficients *a*_3_, *a*_4_, *b*_1_ and *b*_2_ (see Supporting Information), which we neglected in this work, could play an important role in large deformations, such as buckling. Optimal values of these nonlinear elastic coefficients and their uncertainties could be estimated directly from experimental measurements using hierarchical Bayesian uncertainty quantification,^91^ which will be addressed in our future work.

At an even higher pressure of Δ*p* ≈ 58 *k*_B_ *T*_0_/*r*_*c*_^3^ ≈ 71 kPa, the gv undergoes a transition into a two-lobe shape, Figure 7e,h, which is actually closer to the collapse pressure in ref 66. This particular shape was also obtained via a linear buckling analysis by Salahshoor et al.^65^ Both buckling shapes are corroborated by the existence of two low-frequency vibrational modes of similar shape, which are calculated in the next section.

Our buckling simulations were conducted under quasi-static conditions, where pressure was increased slowly to mimic low-frequency ultrasound or static compression experiments. While this approach provides valuable insight into mechanical thresholds, it does not capture the effects of rapid acoustic pressure fluctuations typical of therapeutic ultrasound. Extending the framework to incorporate dynamic loading and cavitation phenomena—potentially through reactive force fields—remains an important direction of our next steps.

### 3.10 Vibrational Modes

Finding the right ultrasound frequency is key for optimal tissue imaging,^135^ as well as for inducing sonophoresis and affecting drug carrier behavior in terms of growth, oscillations, rupture, and drug release via cavitation.^136^ Here, we study the low-frequency modes of gvs that could play a major role in ultrasound backscattering phenomena.

To extract the eigenmodes and their corresponding eigenfrequencies, we run a long simulation of 4000 τ (eq 6, Table S1) of a gv surrounded by dpd water and perform principal component analysis (pca) on the trajectories of the gv vertex beads, see Methods. Some of the extracted low-frequency modes are shown in Figure 8a–f.

**Figure 8:**
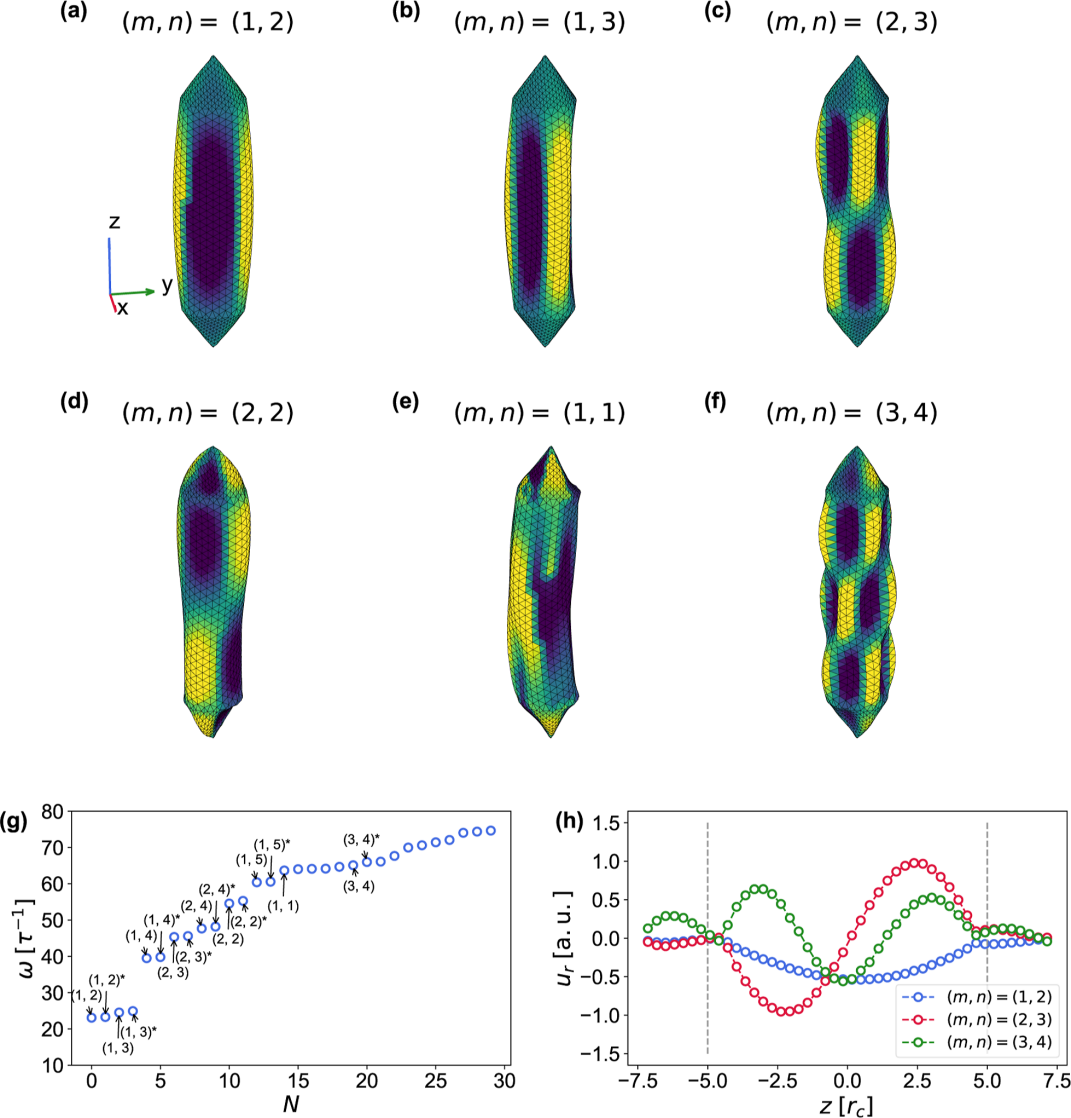
gv vibrational modes. (a–f) Selected low-frequency eigenmodes of a gv with corresponding axial and circumferential numbers *m*, *n*, ordered by increasing frequency. The mesh triangles are colored according to modal displacement. (g) First 30 eigenfrequencies with selected modes labeled by their mode indices (*m*, *n*). The star (*m*,*n*)* denotes an eigenmode rotated by $\frac{\pi }{2n}$ around the *z* axis, relative to the mode with the same indices (*m*, *n*). (h) The *u*_r_(*z*) profile (axial profile) of selected modes. The dashed lines indicate the ends of the gv cylinder.

In the relevant regime, where the gv cylinder is longer than the end cones, the lowest-frequency modes are largely confined to the cylindrical region, as highlighted by Figure 8h. Consequently, the ends of the cylinder impose an effectively rigid boundary condition. In this regime, the modes can be classified not only according to their circumferential number *n*, but also their axial number *m*, on top of their polarization branch, Figure 8a–f. They follow considerably well the solutions to the linearized equations (Donnel theory^137^) of simply supported cylindrical shell (shear diaphragm at both ends at $z=-\frac{1}{2}H_{cyl}^{0}$ and $z=\frac{1}{2}H_{cyl}^{0}$ ),^138^ $u\left(z,\varphi ,t\right)=\left(u_{r}{ê}_{r}+u_{\varphi }{ê}_{\varphi }+u_{z}{ê}_{z}\right)\cos\left(\omega t\right)$ , with

28 $$u_{r}=C_{r}\sin\left(\lambda_{m}z+\frac{m\pi }{2}\right)\cos\left(n\varphi \right)$$

29 $$u_{\varphi }=C_{\varphi }\sin\left(\lambda_{m}z+\frac{m\pi }{2}\right)\sin\left(n\varphi \right)$$

30 $$u_{z}=C_{z}\cos\left(\lambda_{m}z+\frac{m\pi }{2}\right)\cos\left(n\varphi \right)$$

where *u*_*r*_, *u*_φ_, *u*_*z*_ are displacement fields of the cylindrical shell in *r*, φ, and *z* directions, $\lambda_{m}=\frac{m\pi }{H_{cyl}^{0}}$ , and *C*_*r*_, *C*_φ_, *C*_*z*_ are the amplitudes, which depend on *n*, *m*, and the elastic coefficients.^138^ For every mode with *n* ≠ 0, there exists another mode with the same frequency, rotated by $\frac{\pi }{2n}$ around the *z* axis, i.e., with cos­(nφ) and sin­(nφ) in eqs 28–30 interchanged.

The lowest-frequency mode is the doubly degenerate (*m*, *n*) = (1, 2) mode, Figure 8a, with ω ≈ 23.1 τ^–1^, corresponding to frequency ν ≈ 201 MHz. It resembles the gv shape at the second buckling transition, Figure 7e,h.

Our results can be compared with the FEM simulations of gvs in ref 65, where the lowest-frequency vibrational mode is the (*m*, *n*) = (1, 1) mode at ν = 328 MHz. The lower frequency in our case is due to the larger diameter of gv, 140 nm, compared to 85 nm in ref 65. Interestingly, in our case, the (*m*, *n*) = (1, 1) mode appears only as the 15th mode in Figure 8g, with a frequency of ν ≈ 555 MHz.

The elastic shell dynamics that govern these low vibrational modes largely depend on the shell’s elastic parameters, which are independent of the chosen dpd scale. Our chosen time step of Δ*t* = 10^–4^ τ ≈ 6.5 ps is sufficient to resolve frequencies above 10 GHz, well beyond the ultrasound-relevant regime shown in Figure 8g. While our focus is on low-frequency behavior, capturing higher-frequency responses may require smaller scales^139,140^ or coupling with atomistic models.^141^

### 3.11 Rheological Properties: Behavior in Shear Flow

Considering the effects of shear forces on gv dynamics is crucial for their use in targeted drug delivery within the bloodstream. Since gvs are elongated and exhibit a wide range of aspect ratios, their behavior in shear flow can be compared to that of ellipsoids. In shear flow, neutrally buoyant ellipsoids with no-slip boundary conditions undergo rotations known as Jeffery orbits.^142^ During these rotations, the ellipsoid is continuously flipping in the shear plane (the plane spanned by the velocity and its gradient), and the angle θ between its axis and the velocity evolves as

31 $$\tan\left(\theta \right)=\frac{a}{b}\tan\left(\frac{ab\mathrm{\gamma ̇}t}{a^{2}+b^{2}}\right)$$

where *a* and *b* are the major and minor axes of the ellipsoid and γ̇ is the shear rate.

Using obmd, we generate shear flow and apply it to a gv fixed at the center of the domain but free to rotate. In our model, the dissipative interaction between the gv vertex beads and water beads, taking into account eq 9, ensures no-slip boundary condition. This boundary condition leads to a tumbling motion consistent with eq 31, as shown in Figure 9a. In principle, one could functionalize gvs with a hydrophobic surfactant. In such a case, the no-slip boundary condition may no longer apply, potentially altering the qualitative behavior of gvs in shear flow. To investigate this, we explore the gv response in shear flow depending on the repulsive interaction strength *a*_ow_ between the vertices and water beads.

**Figure 9:**
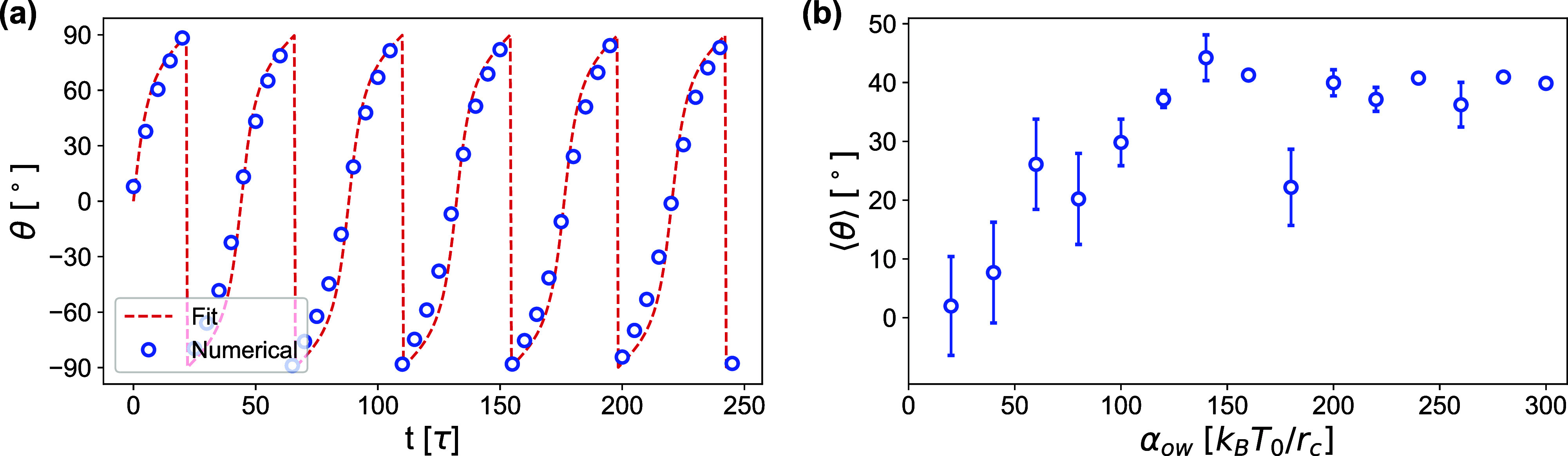
Tumbling and alignment of a gv in shear flow. (a) Time dependence of the gv axis inclination angle θ with respect to the flow direction for gv–water repulsion strength *a*_ow_ = 50 *k*_B_ *T*_0_/*r*_*c*_ and gv–water dissipative interaction strength γ_ow_ = 40 *m*/τ for $\mathrm{\gamma ̇}=0.0862\tau^{-1}$ . Numerical values (blue circles) are compared to eq 31 (red dashed line). (b) Dependence of mean θ on *a*_ow_ for $\mathrm{\gamma ̇}=0.0862\tau^{-1}$ .

The dependence of the mean inclination angle ⟨θ⟩ on *a*_ow_ is shown in Figure 9b. The mean angle and its standard deviation were calculated over the second half of each simulation. For 0 < *a*_ow_ < 50 *k*_B_ *T*_0_/*r*_c_, the gv exhibits tumbling behavior. Above *a*_ow_ ≈ 50 *k*_B_ *T*_0_/*r*_c_, the gv aligns at a fixed angle in the shear plane, which increases to approximately 40° in the limit of large *a*_ow_. Here, γ_ow_ was fixed to the value required by eq 9, which guarantees no-slip boundary conditions for *a*_ow_ = 0.

## 4 Conclusions

The growing demand for encapsulated materials in theranostic ultrasound applications underscores the need for versatile and robust modeling approaches. Current theoretical frameworks often rely on oversimplified descriptions, while fully atomistic models, though detailed, are computationally prohibitive for large-scale or dynamic simulations. In this paper, we developed a general framework for modeling shelled biomaterials, and presented particle-based mesoscopic models of embs and gvs as two representative applications. The elastic energy of their membrane was derived from the continuum theory of elasticity and discretized on a triangular surface, where we drew inspiration from rbc membrane network models. Thus, our description of membrane elasticity builds on the same theoretical foundation as existing force fields and compatibly encompasses previous models, e.g. for rbcs^91,116,143–150^ or viral capsid shells.^151,152^ Moreover, it extends this established framework by incorporating anisotropic elasticity, enabling straightforward application to anisotropic membranes such as gvs, which exhibit orthotropic elasticity due to increased stiffness along the ridges of the GvpA protein.

The elasticity of computational shell models is governed by elastic coefficients, including Young’s moduli, Poisson’s ratios, bending constants, as well as coefficients of mutual influence, which describe the coupling between extensional and shear strains, and Chentsov coefficients, which characterize the interaction of shear strains across different planes. These coefficients can be determined experimentally. Consequently, any membrane composition can be modeled by our framework, as long as its elastic properties are known. This has important implications for fields such as bioengineering and medical applications where elastic properties are concerned, as our framework can model a wide range of membrane-based systems of arbitrary shapes and local anisotropy, from biological cells to artificial capsules.

We validated the framework by simulating stretching, buckling, and shear flow dynamics of embs and gvs, comparing our results with theoretical predictions in the limit of small deformations. The stress–strain curves obtained from the stretching experiments agree well with the theory of linear deformation for both embs and gvs. Furthermore, we were able to reproduce the relationship between the critical buckling pressure and the membrane radius. We also derived analytical expressions that can be used for the experimental determination of the elastic coefficients.

However, the mechanical properties of fluid-immersed objects depend not only on the material of the membrane but also on their environment and the interactions with it. These interactions significantly influence the rheological behavior, which is crucial when modeling membranes in flow. In this study, we focused on simplified, controlled environments to isolate the intrinsic mechanical response of gvs and embs, modeling the surrounding medium as a simple Newtonian fluid within the dpd framework. However, we acknowledge that physiological viscoelastic intracellular conditions can significantly influence the dynamic behavior. Future extensions of the model could incorporate these effects to enhance physiological relevance in gene delivery or imaging scenarios. dpd allows tuning of fluid–object interactions, thereby influencing the dynamic coupling between objects and the fluid. Proper adjustment of the dissipative parameter γ_ow_ enables control over the degree of slip in the boundary condition at the membrane–solvent interface, ranging from no-slip to full slip. Our shear flow numerical experiments demonstrated the rotational periodic motion (flow tumbling) of a single gv suspended in a dpd solvent, consistent with Jeffery’s theoretical predictions. Moreover, we find that both the repulsion and dissipative parameters, *a*_ow_ and γ_ow_, play a crucial role in gv motion, either inducing flow tumbling or leading to flow alignment at a specific inclination angle. This tunability of slip conditions also enables our model to account for physiologically relevant surface modifications, such as lipoprotein adsorption or surfactant functionalization, which may alter interfacial dynamics and influence particle behavior in vivo. While a detailed treatment of membrane functionalization is beyond the scope of this study, our framework is well-suited for future investigations of such effects, offering a path to model vesicle–fluid interactions under more complex biological conditions.

Interaction of ultrasound with encapsulated biomembranes is a highly relevant yet complex topic. While the current work focuses on the mechanical behavior of encapsulated structures under quasi-static conditions, modeling their dynamic response to propagating or standing-wave ultrasound fields is a central objective of our ongoing research. In typical biomedical ultrasound applications (100 kPa–1 MPa, 1–100 MHz), the applied pressures and frequencies are sufficient to induce both linear and nonlinear deformations of ucas, including buckling and collapse, as supported by our results in Figure 6a and Figure 7a. Given that the wavelength of low-frequency ultrasound (10 μm–1 cm) greatly exceeds the characteristic size of the particles (100 nm–1 μm), such wavefields can be reasonably approximated as spatially uniform pressure loads at the particle scale. This approximation justifies the use of isotropic loading in our present simulations as a first-order model of therapeutic ultrasound exposure. Looking ahead, we plan to extend our framework using reactive dissipative particle dynamics (dpd-rx) to explicitly capture ultrasound-driven effects such as bond breakage, shell rupture, and gas exchange under high strain rates. This will allow us to model cavitation-like phenomena and shell failure, ultimately enabling more accurate simulation of therapeutic ultrasound scenarios.

While continuum models focus on modeling the dynamics of individual bubbles to elucidate fundamental principles,^60^ our dpd approach extends beyond these limitations by allowing the simulation of multiple interacting bodies, thus capturing collective behavior and complex interactions that are critical in many applications. The presented modeling framework is a first step toward large-scale simulations of multiple micro- and nanostructures and their interaction with propagating ultrasound waves. Modeling the ultrasound propagation on mesoscopic scales with our virtual us machine^139,140,153,154^ will assist and advance simulation-driven optimization of ultrasound-based theranostics. The optimal parameters that reproduce the given experiments can be efficiently determined using Bayesian uncertainty quantification.^91,118^ The proposed computational framework would allow for controlled testing, data-driven quantification of uncertainties, and rational optimization of experimental us parameters.

## Supplementary Material



## Notes

Elastic energy of embs and gvs with the corresponding nodal forces, numerical validation of the mechanical properties of a rectangular sheet, analytical expressions for the elastic behavior of embs and gvs in the limit of low strain, dependence of the microbubble buckling pressure on its radius, overview of the dissipative particle dynamics (dpd) and simulation details. (PDF)
