# Supplementary material for: Dissipative Particle Dynamics Models of Encapsulated Microbubbles and Nanoscale Gas Vesicles for Biomedical Ultrasound Simulations
Source: ACS Appl Nano Mater. 2025 Aug 4;8(32):16053–70. doi: 10.1021/acsanm.5c02783 (PMC12362338; doi:10.1021/acsanm.5c02783)
Supplement: Supplementary file 1 [file an5c02783_si_001.pdf]

Supporting Information for: Dissipative Particle Dynamics Models of Encapsulated Microbubbles and Nanoscale Gas Vesicles for Biomedical Ultrasound Simulations

Nikolaos Ntarakas,[†,‡] Maša Lah,[†,‡] Daniel Svenšek,[†,‡] Tilen Potisk,[†,‡] and Matej Praprotnik[*,†,‡,¶]

†*Laboratory for Molecular Modeling, National Institute of Chemistry, Hajdrihova 19, SI-1001 Ljubljana, Slovenia*

‡*Department of Physics, Faculty of Mathematics and Physics, University of Ljubljana, Jadranska 19, SI-1000 Ljubljana, Slovenia*

¶*Universitat de Barcelona Institute of Complex Systems (UBICS), C/ Martí i Franqués 1, 08028 Barcelona, Spain*

E-mail: praprot@cmm.ki.si

This PDF file includes:

- Supplementary Figures S1-S10
- Supplementary Tables S1-S3

S1 Elastic energy

Computing the elastic force on a vertex bead of a triangulated object requires an expression for the total elastic energy as a function of the vertex coordinates. The principle of isotropy of space leads to certain restrictions on the form of the elastic energy of anisotropic objects.[1] More specifically, a simultaneous rotation of both the body and the deformation $\boldsymbol{\varepsilon}$ should lead to the same elastic energy U:

$$U(\boldsymbol{\varepsilon}, \mathbf{M}) = U(\mathbf{Q}\boldsymbol{\varepsilon}\mathbf{Q}^T, \mathbf{Q}\mathbf{M}\mathbf{Q}^T) \quad \forall \mathbf{Q} \in \mathrm{O}(3). \tag{S1}$$

The elastic energy U is therefore a scalar isotropic function of two symmetric second order tensors; the elastic strain $\boldsymbol{\varepsilon}$ and the structural tensor $\mathbf{M}$. Conversely one can also view the elastic energy as an anisotropic function with respect to the elastic strain $\boldsymbol{\varepsilon}$:

$$U(\boldsymbol{\varepsilon}, \mathbf{M}) = U(\mathbf{Q}\boldsymbol{\varepsilon}\mathbf{Q}^T, \mathbf{M}) \quad \forall \mathbf{Q} \in \mathcal{G} \subseteq \mathrm{O}(3), \tag{S2}$$

where $\mathcal{G}$ denotes the material symmetry group. The main task is then to find all the possible scalar terms that are invariant under the action of all the group elements $\mathbf{Q} \in \mathcal{G}$.

The elastic energy U can also be derived by first writing down the elastic tensor C^{3D}_{ijkl}, which relates the strain tensor ε^{3D}_{ij} to the elastic stress tensor σ^{3D}_{ij},

$$\sigma^{3D}_{ij} = C^{3D}_{ijkl}\varepsilon^{3D}_{kl}. \tag{S3}$$

In linear elastic theory of thin shells, in-plane and bending deformations are decoupled owing to the assumption that normal stresses are negligible compared to internal tangential stresses (even if bending is induced by normal stress). Under this approximation, the 3D deformation field of the thin shell can be split into in-plane (xy) strain ε_{ij} and bending H_{ij},

$$\varepsilon^{3D}_{ij} = \varepsilon_{ij} - zH_{ij}. \tag{S4}$$

The total elastic energy is then simply a sum of the in-plane elastic (U_{el}) and bending (U_b) contributions:

$$
\begin{aligned}
U &= \int_V \tfrac{1}{2} C^{3D}_{ijkl} \varepsilon^{3D}_{ij} \varepsilon^{3D}_{kl} \, \mathrm{d}V \\
&= \iint_{z=-h/2}^{h/2} \tfrac{1}{2} C^{3D}_{ijkl} \varepsilon_{ij} \varepsilon_{kl} \, \mathrm{d}A \, \mathrm{d}z + \iint_{z=-h/2}^{h/2} \tfrac{1}{2} z^2 C^{3D}_{ijkl} H_{ij} H_{kl} \, \mathrm{d}A \, \mathrm{d}z \\
&= \underbrace{\int \tfrac{1}{2} C_{ijkl} \varepsilon_{ij} \varepsilon_{kl} \, \mathrm{d}A}_{U_{el}} + \underbrace{\int \tfrac{1}{2} D_{ijkl} H_{ij} H_{kl} \, \mathrm{d}A}_{U_b}, \qquad \text{(S5)}
\end{aligned}
$$

where we have identified the two-dimensional in-plane elastic tensor C_{ijkl} and the flexural rigidity tensor D_{ijkl} as integrals of the full 3D elastic tensor C^{3D}_{ijkl} over the thickness h of the shell,

$$
C_{ijkl} = \int_{z=-h/2}^{h/2} C^{3D}_{ijkl} \, \mathrm{d}z = C^{3D}_{ijkl} \, h \qquad \text{(S6)}
$$

$$
D_{ijkl} = \int_{z=-h/2}^{h/2} z^2 C^{3D}_{ijkl} \, \mathrm{d}z = C^{3D}_{ijkl} \, \frac{h^3}{12}. \qquad \text{(S7)}
$$

It should be emphasized that, in general, thin shell theory is not always applicable, particularly for single-molecule layered membranes,[2] and the bending coefficients should, in principle, be regarded as independent of the in-plane elastic ones.

In-plane elastic energy In the constant strain triangle approximation (CST),[3] where the strain field $\boldsymbol{\varepsilon}$ is constant across each of the triangles, in-plane elastic energy U_{el} can be written as a sum over triangles of the triangulated surface,

$$
U_{el} = \int \tfrac{1}{2} C_{ijkl} \varepsilon_{ij} \varepsilon_{kl} \, \mathrm{d}A \to \sum_t \tfrac{1}{2} C^t_{ijkl} \varepsilon^t_{ij} \varepsilon^t_{kl} \, A^t_0, \qquad \text{(S8)}
$$

where the sum runs through all the triangles of the object and the superscript t denotes the corresponding quantity evaluated on the t-th triangle. Since the strains are calculated relative to the reference stress-free configuration, the A^t_0 denotes the stress-free area of the

t-th triangle.

For an isotropic material, the elastic tensor C_{ijkl} is expressed in terms of the Kronecker delta δ_{ij} Eq. (21) in the main text, from which the in-plane elastic energy U_{el}^{iso} is then derived using Eq. (19). However, since isotropic elasticity has been widely used in literature,[2,4] we instead adopt one of the established expressions for the isotropic elastic energy,[5] which is also included in the Mirheo simulation package:[6]

$$U_{el}^{iso} = \frac{K_a}{2}\sum_{t=1}^{N_t} A_t^0[\alpha_t^2 + a_3\alpha_t^3 + a_4\alpha_t^4] + \mu\sum_{t=1}^{N_t} A_t^0[\beta_t + b_1\alpha_t\beta_t + b_2\beta_t^2], \tag{S9}$$

where α_t and β_t are the elastic invariants corresponding to the triangular face t (see Fig S1). This expression also includes nonlinear terms (coefficients $a_3, a_4, b_1,\ b_2$). The invariants α and β are connected with compression and shear deformation, respectively:

$$\alpha = \lambda_1\lambda_2 - 1, \qquad \beta = \frac{\lambda_1^2 + \lambda_2^2}{2\lambda_1\lambda_2} - 1, \tag{S10}$$

with $\lambda_{1,2}$ the eigenvalues of the deformation gradient F_{ij}, corresponding to local principal stretches[2,5] of a deformed triangular element. The nonlinear coefficients a_3, a_4, b_1 and b_2 are often used when describing the elastic properties of RBCs, especially under large strains. In this work, all nonlinear coefficients are set to zero.

For small strains, Eqs. (S8) and (S9) are equivalent, as we show next. We express the eigenvalues $\lambda_{1,2}$ in terms of eigenvalues $e_{1,2}$ of the strain tensor $\boldsymbol{\varepsilon}$, i.e., the principal strains,

and perform a Taylor expansion around zero strain:

$$
\begin{aligned}
\alpha &= \lambda_1\lambda_2 - 1 = \sqrt{1+2e_1}\sqrt{1+2e_2} - 1 \\
&= \sqrt{1 + 2\mathrm{tr}(\boldsymbol{\varepsilon}) + 2(\mathrm{tr}(\boldsymbol{\varepsilon})^2 - \mathrm{tr}(\boldsymbol{\varepsilon}^2))} - 1 \\
&\approx \mathrm{tr}(\boldsymbol{\varepsilon}) + O(|\boldsymbol{\varepsilon}|^2), \qquad \text{(S11)} \\
\beta &= \frac{\lambda_1^2 + \lambda_2^2}{2\lambda_1\lambda_2} - 1 = \frac{1 + 2e_1 + 1 + 2e_2}{2\sqrt{1 + 2(e_1+e_2) + e_1 e_2}} - 1 \\
&= \frac{2 + 2\mathrm{tr}(\boldsymbol{\varepsilon})}{2\sqrt{1 + 2\mathrm{tr}(\boldsymbol{\varepsilon}) + 2(\mathrm{tr}(\boldsymbol{\varepsilon})^2 - \mathrm{tr}(\boldsymbol{\varepsilon}^2))}} - 1 \\
&\approx \mathrm{tr}(\boldsymbol{\varepsilon}^2) - \frac{1}{2}\mathrm{tr}(\boldsymbol{\varepsilon})^2 + O(|\boldsymbol{\varepsilon}|^3). \qquad \text{(S12)}
\end{aligned}
$$

The quantities $\mathrm{tr}(\boldsymbol{\varepsilon})$ and $\mathrm{tr}(\boldsymbol{\varepsilon}^2) - \frac{1}{2}\mathrm{tr}(\boldsymbol{\varepsilon})^2$ are exactly the strain tensor invariants corresponding to area dilatation and pure shear deformations, respectively.

For an orthotropic material, the elastic tensor C_{ijkl} is constructed using a structural tensor $M_{ij} = m_i m_j$, which describes the preferred direction $\mathbf{m}$ (normalized vector), along with the Kronecker delta δ_{ij} (or transverse Kronecker delta $\delta_{ij}^{\perp} = \delta_{ij} - m_i m_j$). The tensor C_{ijkl} should be invariant with respect to the inversion $\mathbf{m} \to -\mathbf{m}$. Taking into account these symmetries one gets four independent terms (Eq. (23) in the main text),

$$
\begin{aligned}
C_{ijkl} &= K_a \delta_{ij}\delta_{kl} + \mu\left(\delta_{ik}\delta_{jl} + \delta_{il}\delta_{jk} - \delta_{ij}\delta_{kl}\right) \\
&\quad + (\mu_L - \mu)(m_i m_l \delta_{jk} + m_j m_l \delta_{ik} + m_i m_k \delta_{jl} + m_j m_k \delta_{il}) \\
&\quad + c\, m_i m_j m_k m_l, \qquad \text{(S13)}
\end{aligned}
$$

with μ_L, c the anisotropic elastic coefficients. Since the GV rotates and deforms over time, the axis $\mathbf{m}$ is updated at each step, simply by tracking the end points of the GV.

Thus, the complete expression for the in-plane elastic energy of the orthotropic material

is

$$U_{el}^{aniso} = U_{el}^{iso} + \sum_{t=1}^{N_t} A_0^t \left[2(\mu_L - \mu) I_3 + \frac{1}{2} c I_4^2 \right], \tag{S14}$$

where we introduced scalar strain invariants for compactness:

$$I_3 = \mathbf{m}^T \boldsymbol{\varepsilon}^T \boldsymbol{\varepsilon} \mathbf{m}, \tag{S15}$$

$$I_4 = \mathbf{m}^T \boldsymbol{\varepsilon} \mathbf{m}. \tag{S16}$$

For vanishing anisotropy, where $\mu_L - \mu = 0$, $c = 0$, isotropic elasticity is recovered in Eq. (S14). In principle, there is one more term quadratic in $\boldsymbol{\varepsilon}$, proportional to $I_5 \equiv \alpha I_4 \approx \mathrm{tr}(\boldsymbol{\varepsilon}) I_4$ or equivalently, a term proportional to $(\delta_{ij} m_k m_l + \delta_{kl} m_i m_j)$ in Eq. (S13). In 2D, it can be expressed as a sum of other invariants in the limit of low strain and was therefore omitted.

In practice, it is sometimes more convenient to work with the so-called engineering constants, which are typically easier to measure and are therefore more frequently reported in experimental literature. They are defined in the small-strain limit through the relations between the strain ε_{ij} and stress σ_{ij} components. For 2D isotropic elasticity the definition is

$$\begin{bmatrix} \varepsilon_{11} \\ \varepsilon_{22} \\ \varepsilon_{12} \end{bmatrix} = \begin{bmatrix} \frac{1}{E} & -\frac{\nu}{E} & 0 \\ -\frac{\nu}{E} & \frac{1}{E} & 0 \\ 0 & 0 & \frac{1}{2G} \end{bmatrix} \begin{bmatrix} \sigma_{11} \\ \sigma_{22} \\ \sigma_{12} \end{bmatrix}, \tag{S17}$$

where E, ν, and $G = \frac{E}{2(1+\nu)}$ are the Young's modulus, Poisson's ratio, and shear modulus, respectively. The bulk K_a and shear μ moduli in Eq. (21) are expressed as

$$K_a = \frac{E}{2(1-\nu)} \tag{S18}$$

$$\mu = G = \frac{E}{2(1+\nu)}, \tag{S19}$$

while the converse relations are:

$$E = \frac{4K_a\mu}{K_a + \mu} \tag{S20}$$

$$\nu = \frac{K_a - \mu}{K_a + \mu}. \tag{S21}$$

In 2D orthotropic elasticity the relation between ε_{ij} and σ_{ij} involves two additional engineering constants:

$$\begin{bmatrix} \varepsilon_{11} \\ \varepsilon_{22} \\ \varepsilon_{12} \end{bmatrix} = \begin{bmatrix} \frac{1}{E_1} & -\frac{\nu_{lt}}{E_1} & 0 \\ -\frac{\nu_{lt}}{E_1} & \frac{1}{E_2} & 0 \\ 0 & 0 & \frac{1}{2G} \end{bmatrix} \begin{bmatrix} \sigma_{11} \\ \sigma_{22} \\ \sigma_{12} \end{bmatrix}, \tag{S22}$$

where $\nu_{lt} = -\frac{\varepsilon_{22}}{\varepsilon_{11}}$ is the Poisson's ratio for uniaxial stress σ_{11}. With that, the other Poisson's ratio $\nu_{tl} = -\frac{\varepsilon_{11}}{\varepsilon_{22}}$ for uniaxial stress σ_{22} is also set,

$$\frac{\nu_{lt}}{E_l} = \frac{\nu_{tl}}{E_t}, \tag{S23}$$

which was already taken into account in Eq. (S22).

The four engineering constants can be used to calculate the elastic coefficients in Eq. (S13):

$$K_a = \frac{E_t E_l (1 + \nu_{lt})}{2(E_l - \nu_{lt}^2 E_t)} \tag{S24}$$

$$\mu = \frac{E_t E_l (1 - \nu_{lt})}{2(E_l - \nu_{lt}^2 E_t)} \tag{S25}$$

$$\mu_L = G \tag{S26}$$

$$c = \frac{E_l^2 + E_l E_t + 4E_t G \nu_{lt}^2 - 4E_l G - 2E_l E_t \nu_{lt}}{E_l - \nu_{lt}^2 E_t}. \tag{S27}$$

The converse relations are

$$E_t = \frac{4\Lambda\mu_L + \Lambda c + 8\mu_L\mu - 4\mu^2 + 2\mu c}{\Lambda + 4\mu_L - 2\mu + c} \tag{S28}$$

$$E_l = \frac{4\Lambda\mu_L + \Lambda c + 8\mu_L\mu - 4\mu^2 + 2\mu c}{\Lambda + 2\mu} \tag{S29}$$

$$\nu_{lt} = \frac{\Lambda}{\Lambda + 2\mu_L} \tag{S30}$$

$$G = \mu_L, \tag{S31}$$

where $\Lambda \equiv K_a - \mu$ is the Lame's first parameter. Thermodynamic stability of the 2D orthotropic material requires $E_t, E_l, G > 0$ and $|\nu_{lt}| < \sqrt{\frac{E_l}{E_t}}$.

Bending energy The bending energy Eq. (24) in the main text is perhaps easier to understand by comparing it to the bending of a flat plate with a particular direction of the anisotropy axis (longitudinal direction), e.g., $\mathbf{m} = \hat{\mathbf{e}}_x$:

$$U_b(\mathbf{m} = \hat{\mathbf{e}}_x, B_{ij} = 0) = \frac{1}{2}D_{xx}H_{xx}^2 + D_{xy}H_{xx}H_{yy} + \frac{1}{2}D_{yy}H_{yy} + \frac{1}{2}D_G H_{xy}^2, \tag{S32}$$

where $D_{xx} = \frac{E_l h^2}{12(1-\nu_{tl}\nu_{lt})}$, $D_{yy} = \frac{E_t h^2}{12(1-\nu_{tl}\nu_{lt})}$, $D_{xy} = \frac{E_t \nu_{lt} h^2}{12(1-\nu_{tl}\nu_{lt})}$ and $D_G = \frac{Gh^2}{12}$ are the flexural rigidities. Clearly, bending along the anisotropy axis x depends on E_l, while bending along y depends on E_t. Given that GVs are stiffer along their axis, one expects circumferential bending to be more easily induced than axial bending. This is also confirmed in our numerical calculations of compression and buckling, as well as in our eigenmode analysis.

There are multiple ways of discretizing the bending energy,[7,8] such as the Monge-form based discretization by Kantor and Nelson,[9] the edge curvature approach by Jülicher,[10] and approaches that discretize the Laplace-Beltrami operator.[11,12] We use the Kantor-Nelson form, which takes into account the angle between each pair of triangles:

$$U_b = \kappa_b \sum_{\langle i,j \rangle} \left[1 - \cos(\theta_{ij} - \theta_{ij}^0)\right], \tag{S33}$$

where κ_b is the bending parameter, while θ_{ij} and θ_{ij}^0 are the actual and spontaneous angles between two adjacent triangles, respectively. To reproduce Eq. (22), $\kappa_b = \frac{2}{\sqrt{3}}\kappa$ must be used. For simplicity, we use Eq. (S33) for the bending energy of both EMBs and GVs. Anisotropic bending effects are retained due to the anisotropic in-plane elasticity generally associated with the bending of shells.

S2 Elastic forces between mesh vertices

Elastic forces acting on the vertex nodes are calculated as derivatives of the total elastic energy $U = U_{el} + U_b$ with respect to the vertex coordinates. In Mirheo,[6] each GPU thread is mapped to one vertex and loops over all adjacent triangles of that vertex. Therefore, only the force on one of the vertices of a given triangle needs to be specified, e.g.,

$$\mathbf{f}_1 = -\frac{\partial U}{\partial \mathbf{x}_1}. \tag{S34}$$

An analytical expression for the deformation gradient F entering U is thus required. The easiest way to calculate the deformation gradient for a CST triangular element is to first super-impose one of the sides of a triangle (e.g., $\boldsymbol{\xi}_{12} = \mathbf{x}_2 - \mathbf{x}_1$) and express the coordinates of the deformed configurations ($\mathbf{x}_2$ and $\mathbf{x}_3$) in terms of their reference counterparts ($\mathbf{x}_2^0$ and $\mathbf{x}_3^0$), see Fig. S1. Here, the basis vectors are $\hat{\mathbf{e}}_1 = \frac{\boldsymbol{\xi}_{12}}{|\boldsymbol{\xi}_{12}|}$ and $\hat{\mathbf{e}}_2 = \frac{\mathbf{n}\times\boldsymbol{\xi}_{12}}{|\mathbf{n}\times\boldsymbol{\xi}_{12}|}$. The deformation gradient then reads[5]

$$\mathsf{F} = \begin{bmatrix} a & b \\ 0 & c \end{bmatrix}, \tag{S35}$$

where a, b, and c are determined by comparing the deformed and reference triangular faces,

see Fig. S1:

$$a = \frac{l}{l_0} = \frac{\sqrt{\boldsymbol{\xi}_{12} \cdot \boldsymbol{\xi}_{12}}}{l_0}, \tag{S36}$$

$$\begin{aligned} b &= \frac{1}{\sin(\phi_0)} \left(\frac{l'}{l'_0} \cos(\phi) - \frac{l}{l_0} \cos(\phi_0) \right) = \frac{1}{\sin(\phi_0)} \left(\frac{\boldsymbol{\xi}_{12} \cdot \boldsymbol{\xi}_{13}}{l l'_0} - a \cos(\phi_0) \right) \\ &= \frac{l_0 l'_0}{2A_0} \left(\frac{\boldsymbol{\xi}_{12} \cdot \boldsymbol{\xi}_{13}}{l l'_0} - a \frac{\boldsymbol{\xi}^0_{12} \cdot \boldsymbol{\xi}^0_{13}}{l_0 l'_0} \right) = \frac{1}{2A_0} \left(\frac{\boldsymbol{\xi}_{12} \cdot \boldsymbol{\xi}_{13}}{a} - a \boldsymbol{\xi}^0_{12} \cdot \boldsymbol{\xi}^0_{13} \right), \end{aligned} \tag{S37}$$

$$c = \frac{l'}{l'_0} \frac{\sin(\phi)}{\sin(\phi_0)} = \frac{\sqrt{(\boldsymbol{\xi}_{12} \times \boldsymbol{\xi}_{13}) \cdot (\boldsymbol{\xi}_{12} \times \boldsymbol{\xi}_{13})}}{l l'_0 \sin(\phi_0)} = \frac{2A}{l l'_0 \sin(\phi_0)} = \frac{A}{a A_0}, \tag{S38}$$

where A denotes the deformed area of the triangle: $A = \frac{1}{2}|\mathbf{n}^*|$, with $\mathbf{n}^* = \boldsymbol{\xi}_{12} \times \boldsymbol{\xi}_{13}$, ϕ_0, ϕ are the angles between the edges $\boldsymbol{\xi}_{12}$ and $\boldsymbol{\xi}_{13}$, l_0, l are the lengths of the edge $\boldsymbol{\xi}_{12}$, while l'_0, l' are the lengths of the edge $\boldsymbol{\xi}_{13}$. The quantities calculated in the reference configuration have a subscript 0. We note that the triangles are indexed so that the vectors $\mathbf{n}^*$ point inside the object.

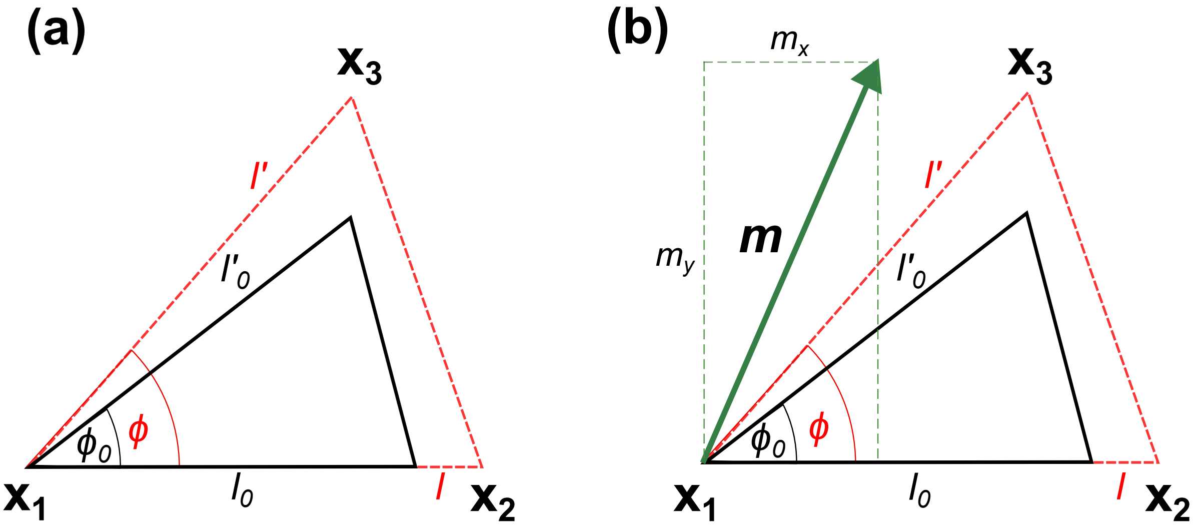

Figure S1: Triangular element deformation and anisotropy axis projection. (a) A deformed triangular face of an object (red dashed lines) superimposed to a reference triangular face (black solid lines) spanned by vertices $\mathbf{x}_1$, $\mathbf{x}_2$ and $\mathbf{x}_3$. b) A scheme representing the projected anisotropy axis $\mathbf{m}$ onto a given triangular element together with its two components m_x and m_y.

Due to the involved expressions for the total force $\mathbf{f}_1$ on a given vertex, we split this force into two contributions; one from the in-plane elastic energy, $\mathbf{f}_1^{el} = -\frac{\partial U^{el}}{\partial \mathbf{x}_1}$ and the other from

the bending energy, $\mathbf{f}_1^b = -\frac{\partial U^b}{\partial \mathbf{x}_1}$. For the orthotropic case, the force $\mathbf{f}_1^{el}$ reads

$$\mathbf{f}_1^{el} = -\frac{\partial U_{el}}{\partial \mathbf{x}_1} = \mathbf{f}_1^{el,iso} - \sum_{t=1}^{N_t} A_0^t \left[2(\mu_L - \mu)\frac{\partial I_3}{\partial \mathbf{x}_1} + cI_4 \frac{\partial I_4}{\partial \mathbf{x}_1} \right], \tag{S39}$$

where I_3, I_4 are the scalar invariants Eqs. (S15)-(S16), and $\mathbf{f}_1^{el,iso}$ denotes the force when only the isotropic part of the energy (Eq. (S9)) is included. We have implemented the orthotropic elastic force as an additional membrane force kernel in Mirheo.

The relevant derivatives with respect to the vertex coordinates, used in numerical computation of Eq. (S39), are:

$$\frac{\partial A}{\partial \mathbf{x}_1} = -\frac{1}{4A}\mathbf{n}^* \times \boldsymbol{\xi}_{32}, \tag{S40}$$

$$\frac{\partial a}{\partial \mathbf{x}_1} = -\frac{\boldsymbol{\xi}_{12}}{ll_0}, \tag{S41}$$

$$\frac{\partial a}{\partial \mathbf{x}_2} = \frac{\boldsymbol{\xi}_{12}}{ll_0}, \tag{S42}$$

$$\frac{\partial a}{\partial \mathbf{x}_3} = 0, \tag{S43}$$

$$\frac{\partial b}{\partial \mathbf{x}_1} = \frac{1}{\sin\phi_0}\left(-\frac{\boldsymbol{\xi}_{13}}{ll_0'} - \frac{\boldsymbol{\xi}_{12}}{ll_0'} + \frac{\boldsymbol{\xi}_{12}\cdot\boldsymbol{\xi}_{13}}{l^3 l_0'}\boldsymbol{\xi}_{12} + \frac{\cos\phi_0}{ll_0}\boldsymbol{\xi}_{12} \right), \tag{S44}$$

$$\frac{\partial b}{\partial \mathbf{x}_2} = \frac{1}{\sin\phi_0}\left(\frac{\boldsymbol{\xi}_{13}}{ll_0'} - \frac{\boldsymbol{\xi}_{12}\cdot\boldsymbol{\xi}_{13}}{l^3 l_0'}\boldsymbol{\xi}_{13} - \frac{\cos\phi_0}{ll_0}\boldsymbol{\xi}_{13} \right), \tag{S45}$$

$$\frac{\partial b}{\partial \mathbf{x}_3} = \frac{1}{\sin\phi_0}\frac{\boldsymbol{\xi}_{12}}{ll_0'}, \tag{S46}$$

$$\frac{\partial c}{\partial \mathbf{x}_1} = \frac{\mathbf{n}^* \times \boldsymbol{\xi}_{23}}{2All_0'\sin\phi_0} + \frac{2A}{l^3 l_0'\sin\phi_0}\boldsymbol{\xi}_{12}, \tag{S47}$$

$$\frac{\partial c}{\partial \mathbf{x}_2} = \frac{\mathbf{n}^* \times \boldsymbol{\xi}_{13}}{2All_0'\sin\phi_0} - \frac{2A}{l^3 l_0'\sin\phi_0}\boldsymbol{\xi}_{12}, \tag{S48}$$

$$\frac{\partial c}{\partial \mathbf{x}_3} = \frac{\mathbf{n}^* \times \boldsymbol{\xi}_{12}}{2All_0'\sin\phi_0}. \tag{S49}$$

The invariants in Eq. (S39) and their derivatives with respect to the vertex coordinate can be more compactly expressed using the invariants involving the axis of anisotropy $\mathbf{m}$

and the right Cauchy strain tensor C

$$I_3^C = \mathbf{m}^T \mathsf{C}^T \mathsf{C} \mathbf{m} = 4I_3 + 4I_4 + 1 = m_x^2(a^4 + a^2b^2) + 2m_x m_y(a^3 b + ab^3 + abc^2) + m_y^2(a^2b^2 + (b^2 + c^2)^2), \tag{S50}$$

$$I_4^C = \mathbf{m}^T \mathsf{C} \mathbf{m} = 2I_4 + 1 = m_x^2 a^2 + 2m_x m_y ab + m_y^2(b^2 + c^2), \tag{S51}$$

where

$$\mathsf{C} = \mathsf{F}^T \mathsf{F} = \begin{bmatrix} a^2 & ab \\ ab & b^2 + c^2 \end{bmatrix}. \tag{S52}$$

The derivatives of the invariants I_3^C and I_4^C are:

$$\frac{\partial I_3^C}{\partial \mathbf{x}_1} = m_x^2\left(4a^3 \frac{\partial a}{\partial \mathbf{x}_1} + 2ab^2 \frac{\partial a}{\partial \mathbf{x}_1} + 2a^2 b \frac{\partial b}{\partial \mathbf{x}_1}\right) + 2m_x m_y \frac{\partial a}{\partial \mathbf{x}_1}\left(3a^2 b + b^3 + bc^2\right) + 2m_x m_y \frac{\partial b}{\partial \mathbf{x}_1}\left(a^3 + 3ab^2 + ac^2\right) + 4m_x m_y \frac{\partial c}{\partial \mathbf{x}_1} abc + 2m_y^2 \frac{\partial a}{\partial \mathbf{x}_1} ab^2 + m_y^2 \frac{\partial b}{\partial \mathbf{x}_1}(2a^2 b + 4(b^2 + c^2)b) + 4m_y^2 \frac{\partial c}{\partial \mathbf{x}_1}(b^2 + c^2)c, \tag{S53}$$

$$\frac{\partial I_4^C}{\partial \mathbf{x}_1} = 2m_x^2 a \frac{\partial a}{\partial \mathbf{x}_1} + 2m_x m_y b \frac{\partial a}{\partial \mathbf{x}_1} + 2m_x m_y a \frac{\partial b}{\partial \mathbf{x}_1} + 2m_y^2 \frac{\partial b}{\partial \mathbf{x}_1} b + 2m_y^2 \frac{\partial c}{\partial \mathbf{x}_1} c. \tag{S54}$$

The vector $\mathbf{m}$ is defined locally for each triangle and is written in the same basis as the deformation gradient (Eq. (S35)). This vector is derived from the main axis of the gas vesicle $\mathbf{m}^*$, which is for simplicity calculated from the positions of the tips of the conical ends $\mathbf{v}_t$ and $\mathbf{v}_b$,

$$\mathbf{m}^* = \mathbf{v}_t - \mathbf{v}_b, \tag{S55}$$

where the two choices of assigning $\mathbf{v}_t$ and $\mathbf{v}_b$ are equivalent due to the $\mathbf{m}^* \to -\mathbf{m}^*$ symmetry. We project $\mathbf{m}^*$ onto the triangle by subtracting the component along the triangle unit normal

$\mathbf{n}$:

$$\mathbf{m}^{\|} = \mathbf{m}^* - \mathbf{n}(\mathbf{n} \cdot \mathbf{m}^*), \tag{S56}$$

$$\mathbf{n} = \frac{\boldsymbol{\xi}_{12} \times \boldsymbol{\xi}_{13}}{|\boldsymbol{\xi}_{12} \times \boldsymbol{\xi}_{13}|}. \tag{S57}$$

The components of the projected vector $\mathbf{m}^{\|}$ in the basis of the deformation gradient Eq. (S35), then read

$$m_x = \frac{\mathbf{m}^{\|} \cdot \boldsymbol{\xi}_{12}}{|\mathbf{m}^{\|}||\boldsymbol{\xi}_{12}|}, \tag{S58}$$

$$m_y = \frac{\mathbf{m}^{\|} \cdot (\mathbf{n} \times \boldsymbol{\xi}_{12})}{|\mathbf{m}^{\|}||\mathbf{n} \times \boldsymbol{\xi}_{12}|}. \tag{S59}$$

Calculating the bending forces on the vertices requires iterating through all the pairs of triangles of the triangulated mesh, see Fig. S2. This is a consequence of the Kantor-Nelson discretization of the bending energy Eq. (S33), which takes into account the angles θ_{ij} between the pairs of adjacent triangles i and j. The nodal bending forces on all the vertices $\mathbf{x}_1, \mathbf{x}_2, \mathbf{x}_3, \mathbf{x}_4$ of two adjacent triangles read[13]

$$\mathbf{f}_1^b = -\frac{\partial U_b}{\partial \mathbf{x}_1} = b_{11}\boldsymbol{\xi} \times \boldsymbol{\xi}_{32} + b_{12}\left(\boldsymbol{\xi} \times \boldsymbol{\xi}_{43} + \boldsymbol{\zeta} \times \boldsymbol{\xi}_{32}\right) + b_{22}\boldsymbol{\zeta} \times \boldsymbol{\xi}_{43}, \tag{S60}$$

$$\mathbf{f}_2^b = -\frac{\partial U_b}{\partial \mathbf{x}_2} = b_{11}\boldsymbol{\xi} \times \boldsymbol{\xi}_{13} + b_{12}\boldsymbol{\zeta} \times \boldsymbol{\xi}_{13}, \tag{S61}$$

$$\mathbf{f}_3^b = -\frac{\partial U_b}{\partial \mathbf{x}_3} = b_{11}\boldsymbol{\xi} \times \boldsymbol{\xi}_{21} + b_{12}\left(\boldsymbol{\xi} \times \boldsymbol{\xi}_{14} + \boldsymbol{\zeta} \times \boldsymbol{\xi}_{21}\right) + b_{22}\boldsymbol{\zeta} \times \boldsymbol{\xi}_{14}, \tag{S62}$$

$$\mathbf{f}_4^b = -\frac{\partial U_b}{\partial \mathbf{x}_4} = b_{11}\boldsymbol{\xi} \times \boldsymbol{\xi}_{31} + b_{12}\boldsymbol{\zeta} \times \boldsymbol{\xi}_{31}, \tag{S63}$$

where $b_{11} = -\beta_b \cos(\theta_{ij})/|\boldsymbol{\xi}|^2$, $b_{12} = \beta_b/(|\boldsymbol{\xi}||\boldsymbol{\zeta}|)$, $b_{22} = -\beta_b \cos(\theta_{ij})/|\boldsymbol{\zeta}|^2$ and $\beta_b = \kappa_b \left(\sin\theta_{ij} \cos\theta_{ij}^0 - \cos\theta_{ij} \sin\theta_{ij}^0\right)$. The normals of the triangles are $\boldsymbol{\xi} = \boldsymbol{\xi}_{12} \times \boldsymbol{\xi}_{13}$ and $\boldsymbol{\zeta} = \boldsymbol{\xi}_{41} \times \boldsymbol{\xi}_{43}$, see Fig. S2. The cosine of the angle between the adjacent triangles is determined from the scalar products of the normals, $\cos\theta_{ij} = \frac{\boldsymbol{\xi} \cdot \boldsymbol{\zeta}}{|\boldsymbol{\xi}||\boldsymbol{\zeta}|}$, which leads to $\sin\theta_{ij} = \pm\sqrt{1 - \cos^2\theta_{ij}}$, where the positive sign is taken if $(\boldsymbol{\xi} - \boldsymbol{\zeta}) \cdot \boldsymbol{\xi}_{24} > 0$ and negative otherwise.

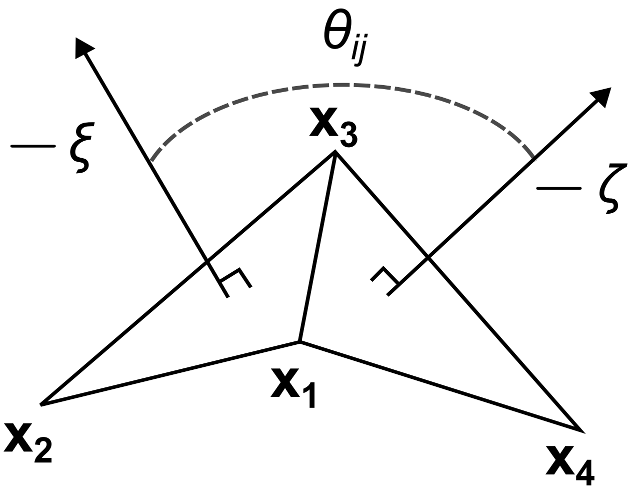

Figure S2: Vertices, normals $\boldsymbol{\xi}$ and $\boldsymbol{\zeta}$, and the angle θ_{ij} between the adjacent triangles for the calculation of bending forces. For clarity, the inverse normals are shown. Note the interchange of $\mathbf{x}_1$ and $\mathbf{x}_2$ between our indexing convention and that of Ref.[13] In our convention, the normals point inward.

S3 Mechanical properties - technical details

S3.1 Numerical validation of elastic properties

To validate that the elastic membranes of the ultrasound contrast agents reproduce the desired elastic properties, such as Young's moduli and Poisson's ratios, we test the behavior of a rectangular sheet under extensional stresses σ_{xx} and σ_{yy} and measure their strains ε_{xx} and ε_{yy}. To measure the shear modulus G, we impose a shear strain and measure the resulting shear stress response.

In these validation simulations, the axis of anisotropy is $\mathbf{m} = \hat{\mathbf{e}}_x$, and the engineering constants are set to $E_t = 50\, k_B T_0/r_c^2$, $E_l = 250\, k_B T_0/r_c^2$, $\nu_{lt} = 0.8$, $G = 5.7\, k_B T_0/r_c^2$.

As seen in Fig. S3, the measured Young's moduli, Poisson's ratios ν_{lt} and ν_{tl}, and shear modulus μ_L agree excellently with the simulation input values. To calculate the Poisson's ratio, we measure $\nu_{lt} = -\frac{\varepsilon_{yy}}{\varepsilon_{xx}}$ for stretching forces along the x axis, as well as its reciprocal equivalent $\nu_{tl} = -\frac{\varepsilon_{xx}}{\varepsilon_{yy}}$ for stretching along the y axis. The two Poisson's ratios are related according to Eq. (S23), yielding $\nu_{tl} = 0.16$.

The corresponding in-plane displacement fields $u_x(x, y)$ of the rectangular sheet are shown

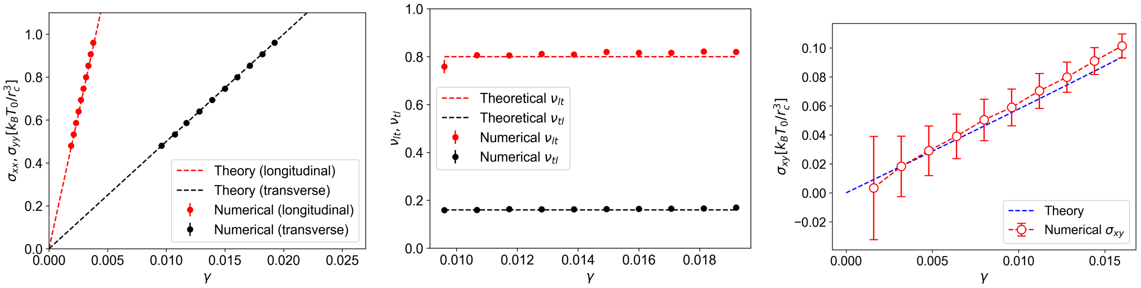

Figure S3: Stretching and shearing of a rectangular sheet. (a) Dilatational stresses σ_{xx} and σ_{yy} as functions of strain when stretching the sheet along the x and y axis. (b) Poisson's ratios ν_{lt} and ν_{tl} as a function of strain. (c) Shear stress σ_{xy} as a function of shear strain. Dashed lines represent theoretical values, set as simulation parameters, and points represent their measured values.

for different values of dilatational stress, Fig. S4, and shear strain, Fig. S5.

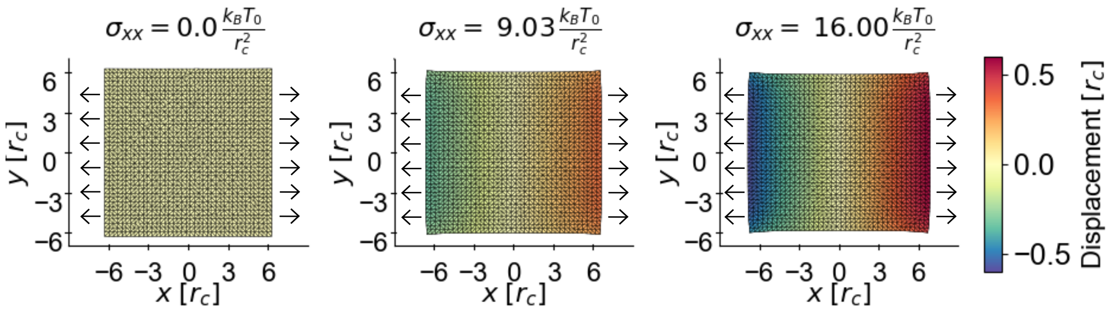

Figure S4: In-plane displacement field $u_x(x, y)$ for different values of dilatational stress σ_{xx}. $u_x(x, y)$ is displayed for the rectangular sheet, stretched along the x axis. The arrows denote the directions of the forces on the leftmost and rightmost vertices of the sheet.

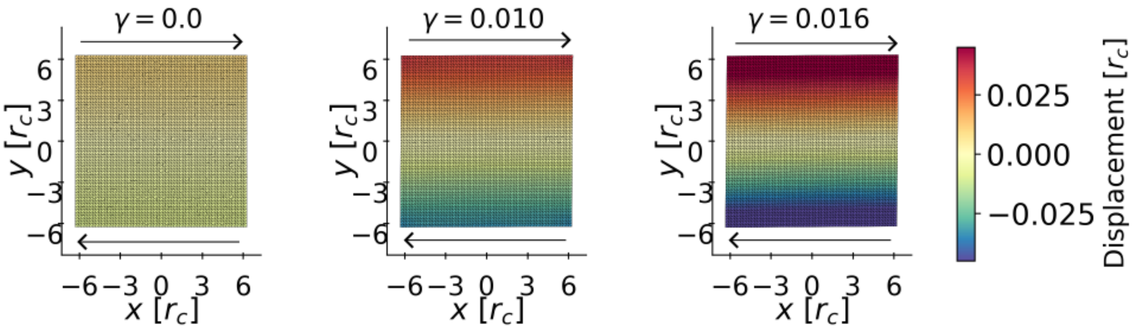

Figure S5: $u_x(x, y)$ for different values of shear strain γ. The arrows indicate the directions of displacement of the topmost and bottommost vertices of the sheet.

S3.2 Microbubbles

Stretching Stretching of a microbubble results in an increased diameter along the stretching direction and a decrease of the diameter perpendicular to the stretching direction. The stretching is experimentally induced by attaching two micron-sized spherical silica beads at the ends of a given object and displacing them in opposite directions using lasers. The equilibrium condition reads:[2]

$$\frac{\sigma_{\theta\theta}}{R_\theta} + \frac{\sigma_{\varphi\varphi}}{R_\varphi} = 0, \tag{S64}$$

where R_θ and R_φ are the principal radii of curvature along the meridian and equator, respectively. In the low strain limit $R_\theta \approx R_\varphi$, which yields $\sigma_{\varphi\varphi} = -\sigma_{\theta\theta}$. The tangential stress at the equator can be calculated from the total force F_{tot}: $\sigma_{\theta\theta} = \frac{F_{tot}}{2\pi R}$, which yields the strain along the equator $\varepsilon_{\varphi\varphi} = \frac{F_{tot}}{4\pi R\mu}$. Taking into account the relation $\varepsilon_{\varphi\varphi} = \frac{D-D_0}{D_0}$, the diameter then reads:

$$D = D_0 - \frac{F_{tot}}{2\pi\mu}. \tag{S65}$$

Compression Compression is achieved by exerting a uniform excess pressure Δp on the fluid containing EMBs. The induced elastic stresses within the spherical EMB shell are homogeneous and can be determined by virtually halving the sphere and calculating the total force exerted by the pressure difference on the hemisphere, see Fig. 4 in the main text. This force equals $\Delta p \pi R^2$, which induces a uniform stress $\sigma_{\theta\theta} = \sigma_{\varphi\varphi} \equiv \sigma$ within the shell:

$$\sigma = -\frac{\Delta p \pi R^2}{2\pi R} = -\frac{R}{2}\Delta p. \tag{S66}$$

The relative EMB volume change is $\frac{\Delta V}{V} = 3\frac{\Delta R}{R} = 3\varepsilon_{\theta\theta} = 3\varepsilon_{\varphi\varphi}$, or also $\frac{\Delta V}{V} = \frac{3}{2}\frac{\Delta S}{S}$, where $\frac{\Delta S}{S}$ is the relative EMB surface area change. Taking into account

$$\varepsilon_{\theta\theta} = \frac{\sigma_{\theta\theta}}{E} - \frac{\nu\sigma_{\varphi\varphi}}{E} \tag{S67}$$

$$\varepsilon_{\varphi\varphi} = -\frac{\nu\sigma_{\theta\theta}}{E} + \frac{\sigma_{\varphi\varphi}}{E}, \tag{S68}$$

or directly the definition of 2D compression

$$\frac{\Delta S}{S} = \frac{1}{K_a}\frac{\mathrm{tr}(\boldsymbol{\sigma})}{2}, \tag{S69}$$

with $\mathrm{tr}(\boldsymbol{\sigma}) = \sigma_{\theta\theta} + \sigma_{\varphi\varphi}$, one arrives at

$$\frac{\Delta V}{V} = \frac{3(1-\nu)}{E}\sigma = \frac{3}{2K_a}\sigma \tag{S70}$$

and with Eq. (S66) finally at the relative EMB volume change

$$\frac{\Delta V}{V} = -\frac{3(1-\nu)R}{2E}\Delta p = -\frac{3R}{4K_a}\Delta p. \tag{S71}$$

For an orthotropic elastic sphere, the result Eq. (S70) becomes

$$\frac{\Delta V}{V} = \frac{1}{E_l}\left(1 - 3\nu_{lt} + 2\frac{E_l}{E_t}\right)\sigma, \tag{S72}$$

which reduces to Eq. (S70) when $E_l = E_t = E$.

Buckling Critical buckling pressure of an EMB scales inversely with the square of its radius:[14]

$$\Delta p_c = C\frac{2E^{3D}}{\sqrt{3(1-\nu^2)}}\left(\frac{h}{R}\right)^2, \tag{S73}$$

where C is a dimensionless empirical factor, which in real experiments is often found to be less than 1 owing to the various imperfections of the shell. We find that the Eq. (S73) is in excellent agreement with the simulations, Fig. S6, with $C = 0.52 \pm 0.01$.

S3.3 Gas vesicles

Stretching When stretching the GV along the axis one can estimate the local stress tensor in the cylindrical part from the total force: $\sigma_{zz} = \frac{F_{tot}}{2\pi R}$. The only other component is

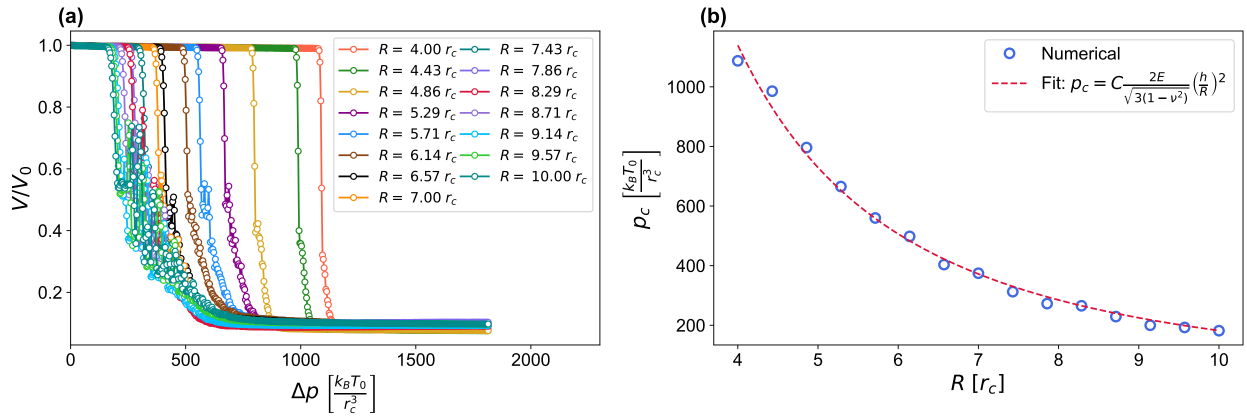

Figure S6: EMB buckling pressure. (a) Volume of an EMB as a function of external pressure Δp for various EMB sizes R. (b) Buckling pressure dependence on R, fitted (dashed red) with Eq. (S73).

the circumferential stress $\sigma_{\varphi\varphi}$, which is zero, $\sigma_{\varphi\varphi} = 0$. For the resulting longitudinal and circumferential strain we therefore find rather simple relations:

$$\varepsilon_{zz} = \frac{1}{E_l}\frac{F_{tot}}{2\pi R} \tag{S74}$$

$$\varepsilon_{\varphi\varphi} = -\frac{\nu_{lt}}{E_l}\frac{F_{tot}}{2\pi R}. \tag{S75}$$

As has been done in the main text, one can then use these relations to estimate the longitudinal Young's modulus and the Poisson's ratio ν_{lt}.

Torsion On the other hand, to extract the shear modulus G, one has to induce shear strain on the membrane. We apply this strain by rotating the ends of the GV in opposite directions by an equal angle of $\theta/2$. The torsional strain is therefore $\gamma = \frac{R_0\theta}{H^0_{cyl}}$. The torsional stress is calculated from the measured forces on the vertices, which are needed to sustain this strain. As one can see from Fig. S7, the theoretical dependence $\sigma_{\varphi r} = G\gamma$ matches the numerical calculations excellently.

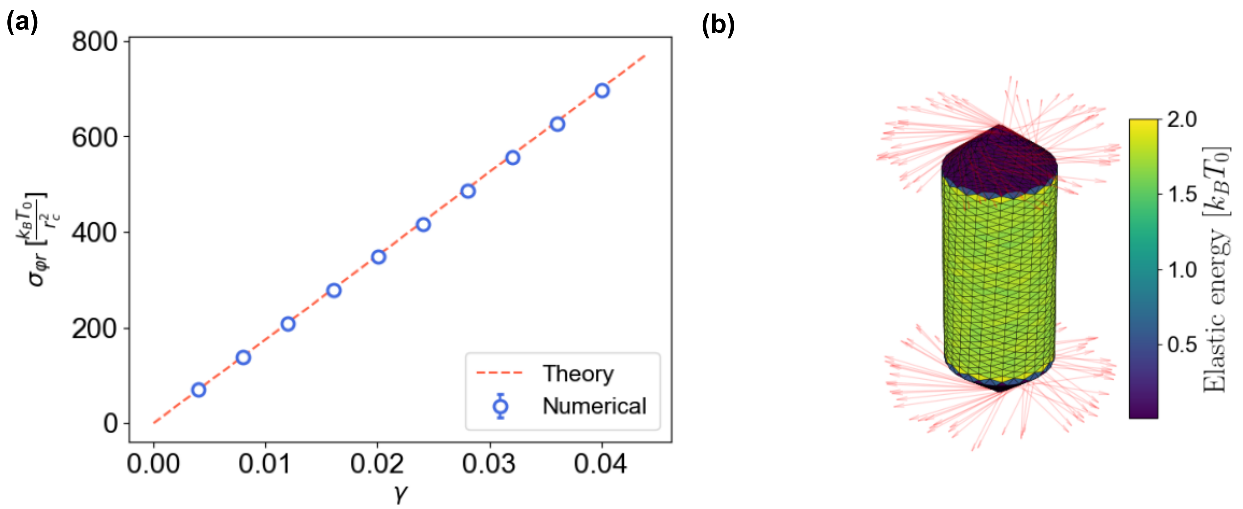

Figure S7: GV torsion. (a) Torsional stress $\sigma_{\varphi r}$ as a function of the torsional strain $\gamma = \frac{R\theta}{H^0_{cyl}}$ for a GV twisted around the main axis. The numerical values are compared to theoretical values in the limit of low strain. (b) Side view of a GV under a specific torsional strain. The vertices are colored according to their total elastic energy.

Compression Similarly one can obtain an equation for the volume change of GVs:

$$\varepsilon_{zz} = \frac{\sigma_{zz}}{E_l} - \frac{\nu_{tl}\sigma_{\varphi\varphi}}{E_t} = \frac{\sigma_{zz}}{E_l} - \frac{\nu_{lt}\sigma_{\varphi\varphi}}{E_l} \tag{S76}$$

$$\varepsilon_{\varphi\varphi} = \frac{\sigma_{\varphi\varphi}}{E_t} - \frac{\nu_{lt}\sigma_{zz}}{E_l}, \tag{S77}$$

where $\sigma_{zz} = \frac{1}{2}\Delta p R_0$, $\sigma_{\varphi\varphi} = \Delta p R_0$, and we used Eq. (S23). For cylindrical shapes, the volume change is $\frac{\Delta V}{V_0} = \varepsilon_{zz} + 2\varepsilon_{\varphi\varphi}$, which yields:

$$\frac{\Delta V}{V_0} = -\frac{\Delta p R_0}{2E_l}\left(1 - 4\nu_{lt} + 4\frac{E_l}{E_t}\right). \tag{S78}$$

S4 Dissipative Particle Dynamics (DPD)

S4.1 Weight functions

In DPD, the force between the particles linearly decreases with interparticle distance $r_{ij} = |\mathbf{r}_{ij}|$ up to a cutoff $r_{ij} = r_c$. This is encompassed in the conservative weight function ω_C in Eq. (1):

$$\omega_C(r_{ij}) = \begin{cases} 1 - \frac{r_{ij}}{r_c}, & r_{ij} < r_c \\ 0, & r_{ij} \geq r_c \end{cases}, \tag{S79}$$

To satisfy the fluctuation-dissipation theorem, the weight functions of the random and dissipative forces (Eqs. (2)-(3)) are related through $\omega_R^2 = \omega_D$. Similarly, the amplitude of the random force $\sigma_{\alpha\beta}$ is fixed by temperature T_0 and the friction parameter $\gamma_{\alpha\beta}$ to $\sigma_{\alpha\beta}^2 = 2k_B T_0 \gamma_{\alpha\beta}$.

At a given temperature, the viscosity of the DPD fluid is determined mainly by the dissipative parameter $\gamma_{\alpha\alpha}$ in Eq. (2) and the density ρ. To allow additional control of viscosity and the Schmidt number (Eq. (S85)), several authors[15,16] have modified the dissipative force kernel ω_D in Eq. (2). We use the form presented in,[15] which is also commonly used in the red blood cell (RBC) modeling community:[4,17–19]

$$\omega_D(r_{ij}) = \begin{cases} \left(1 - \frac{r_{ij}}{r_c}\right)^{2k}, & r_{ij} < r_c \\ 0, & r_{ij} \geq r_c \end{cases}, \tag{S80}$$

where k is the so-called kernel power.

S4.2 Fundamental scales, simulation parameters and details

The EMB and GV sets of fundamental scales, described in the Methods section, are given in Table S1. The simulation timestep, typical simulation box sizes and OBMD parameters used in shear flow simulations are given in Table S2.

Table S1: Fundamental scales r_c, ε, m, and τ for the EMB and GV unit sets, along with the elastic coefficients down-scaling factor f_{scale}; $T_0 = 300\,\mathrm{K}$.

Fundamental scale	Value (EMB unit set)	Value (GV unit set)
Length scale r_c	$0.25\,\mu\mathrm{m}$	$35\,\mathrm{nm}$
Energy scale ε	$k_B T_0$	$k_B T_0$
Mass scale m	$5.19 \times 10^{-18}\,\mathrm{kg}$	$1.42 \times 10^{-20}\,\mathrm{kg}$
Time scale τ	$8.85 \times 10^{-6}\,\mathrm{s}$	$6.49 \times 10^{-8}\,\mathrm{s}$
Scaling factor f_{scale}	0.0074	0.079

Table S2: Values of the simulation parameters used, unless stated otherwise.

Simulation parameter	Symbol	Value
Integration timestep	Δt	$0.0001\,\tau$
Box size - mechanical simulations	$L_x \times L_y \times L_z$	$25\,r_c \times 25\,r_c \times 25\,r_c$
Box size - shear flow simulations	$L_x \times L_y \times L_z$	$50\,r_c \times 25\,r_c \times 25\,r_c$
Buffer mass control parameter	α_B	0.6
Buffer relaxation time	τ_B	$10\Delta t$
Buffer length	x_B	$7.5\,r_c$

To integrate the particle dynamics, we use a modified velocity-Verlet algorithm:[20]

$$\mathbf{r}_k(t+\Delta t) = \mathbf{r}_k(t) + \Delta t \mathbf{v}_k(t) + \frac{(\Delta t)^2}{2m_k}\mathbf{f}_k(t) \tag{S81}$$

$$\tilde{\mathbf{v}}_k(t+\Delta t) = \mathbf{v}_k(t) + \lambda \frac{\Delta t}{m_k}\mathbf{f}_k(t) \tag{S82}$$

$$\mathbf{f}_k(t+\Delta t) = \mathbf{f}_k\left(\mathbf{r}_k(t+\Delta t), \tilde{\mathbf{v}}_k(t+\Delta t)\right) \tag{S83}$$

$$\mathbf{v}_k(t+\Delta t) = \mathbf{v}_k(t) + \frac{\Delta t}{2m_k}\left(\mathbf{f}_k(t) + \mathbf{f}_k(t+\Delta t)\right), \tag{S84}$$

with $\lambda = 1/2$, and $m_k, \mathbf{r}_k, \mathbf{v}_k$, $\mathbf{f}_k$ the mass, position vector, velocity, and total force acting on the k-th DPD bead.

S4.3 DPD interactions

The DPD interactions, described by $a_{\alpha\beta}$ and $\gamma_{\alpha\beta}$ in Eqs. (1)-(2), are given in Table 1 in the main text. These parameters are set to approximately reproduce the viscosity of the water and gas phases, as well as the no-slip boundary condition between the shell surface and the phases. To characterize the water and gas phases, we calculate the shear viscosity η, the self-diffusion constant D, and the Schmidt number

$$Sc = \frac{\eta}{\rho D} \tag{S85}$$

for a relevant range of DPD parameters $a_{\alpha\alpha}$ and $\gamma_{\alpha\alpha}$, see Fig. S8. We choose different values of parameters γ_{ww} and γ_{gg} depending on the length scale (Table S1) and the actual (scaled-down) viscosities of water and gas phases.

The physical and corresponding model (scaled-down) parameters for GVs and EMBs used in our simulations are summarized in Table S3. Most of the physical parameters, except for the shear modulus μ_L in GVs, which was estimated by its isotropic counterpart μ, were obtained from available literature.[21–23]

S4.4 Inducing pressure

We induce pressure by increasing the interaction parameter between the water beads a_{ww}. Here, we verify that such a protocol leads to a pressure, which matches nicely to the semi-empirical equation of state Eq. (5) as seen in Fig. S9.

S4.5 Shear flow

Shear flow is induced by adding a term that applies equally opposite forces to the buffers. This term, given in Eq. (12) in the main text consists of shear stress P_{yx}, and a unit vector $\hat{\mathbf{t}}$ ($\hat{\mathbf{t}} = \hat{\mathbf{e}}_y$ at the right buffer and $\hat{\mathbf{t}}$ = -$\hat{\mathbf{e}}_y$ at the left buffer).

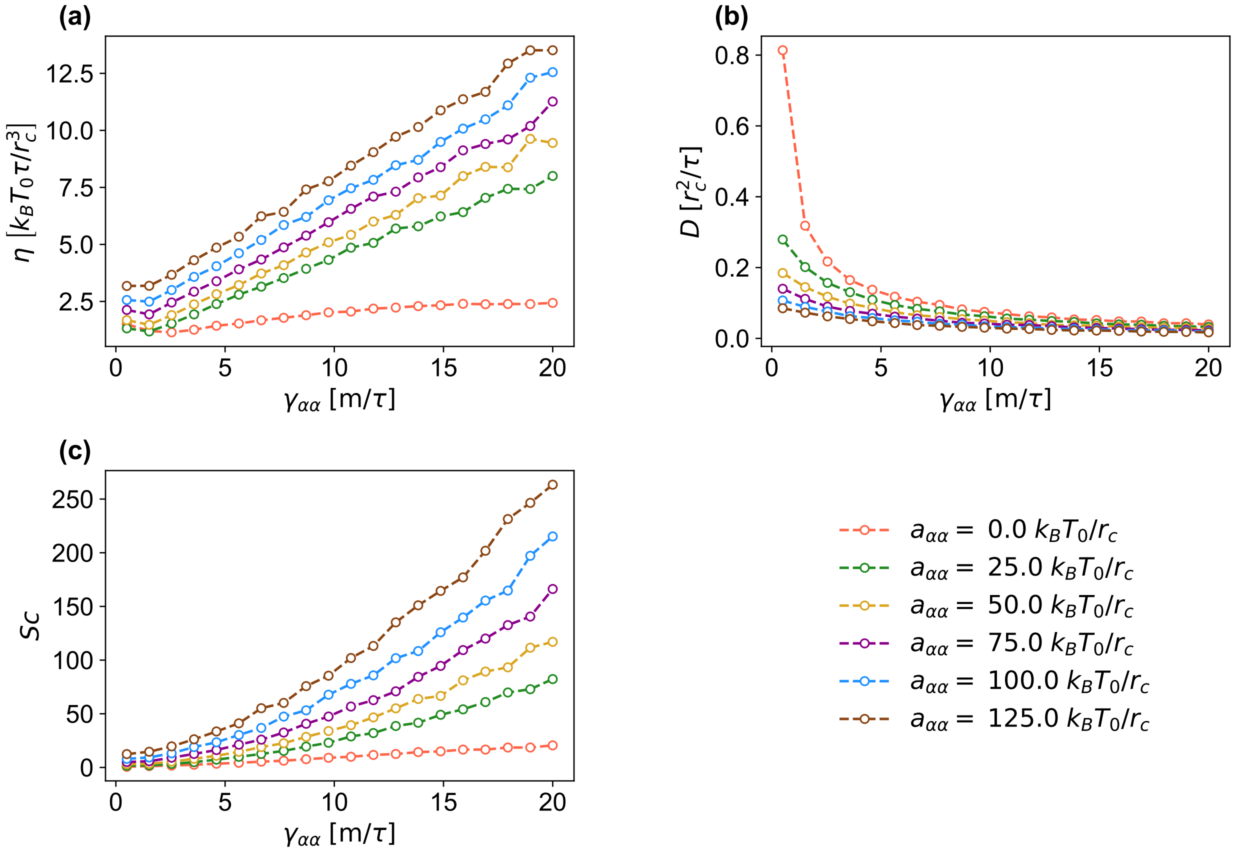

Figure S8: Rheological properties of DPD water. (a) Shear viscosity, (b) self-diffusion constant D and (c) Schmidt number Sc as a function of $\gamma_{\alpha\alpha}$ at different values of the interaction parameter $a_{\alpha\alpha}$.

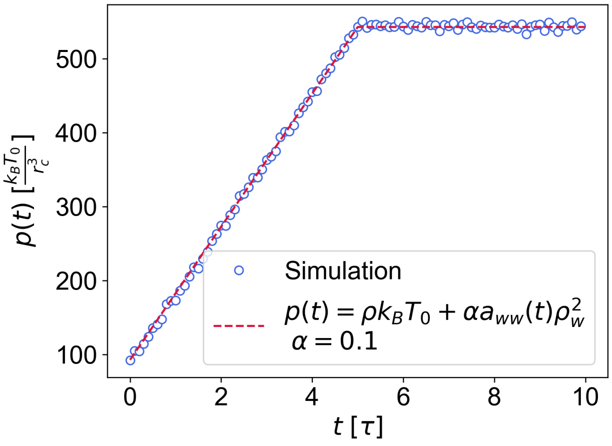

Figure S9: Inducing time dependent pressure. Measured simulation pressure over time (blue circles) compared with pressure from the semi-empirical equation of state (red dashed line).

Table S3: Physical (above dashed line) and model 2D (below dashed line) parameters for GVs and EMBs. The 2D elastic moduli are downscaled by the scaling factor f_{scale}.

Parameter	Model value	Physical value
**Gas vesicles**[21]		
$H = H_{cyl} + 2H_{cone}$ GV height	$14.3\, r_c$	$500\,\mathrm{nm}$
H_{cone} GV cone height	$2.1\, r_c$	$75\,\mathrm{nm}$
D_0 diameter	$4\, r_c$	$140\,\mathrm{nm}$
h shell thickness	$0.057\, r_c$	$2\,\mathrm{nm}$
ρ_s shell mass density	$3.97\, m\, r_c^{-3}$	$1320\,\mathrm{kg\,m^{-3}}$
ρ_{gas} gas mass density	$1.12 \times 10^{-2}\, m\, r_c^{-3}$	$3.72\,\mathrm{kg\,m^{-3}}$
E_t^{3D} transverse Young's modulus	$1.04 \times 10^{7}\, k_B T_0\, r_c^{-3}$	$1.0\,\mathrm{GPa}$
E_l^{3D} longitudinal Young's modulus	$4.14 \times 10^{7}\, k_B T_0\, r_c^{-3}$	$4.0\,\mathrm{GPa}$
ν_{lt} Poisson's ratio	0.3	0.3
K_a bulk modulus	$3.11 \times 10^{4}\, k_B T_0\, r_c^{-2}$	$1.05 \times 10^{-1}\,\mathrm{Nm^{-1}}$
μ shear modulus	$1.67 \times 10^{4}\, k_B T_0\, r_c^{-2}$	$5.66 \times 10^{-2}\,\mathrm{Nm^{-1}}$
μ_L shear modulus	$1.67 \times 10^{4}\, k_B T_0\, r_c^{-2}$	$5.66 \times 10^{-2}\,\mathrm{Nm^{-1}}$
c anisotropic stiffness	$1.43 \times 10^{5}\, k_B T_0\, r_c^{-2}$	$4.85 \times 10^{-1}\,\mathrm{Nm^{-1}}$
κ_C bending parameter	$16.14\, k_B T_0$	$6.68 \times 10^{-20}\, J$
**Protein-based microbubbles**[22,23]		
R_0 radius	$4\, r_c$	$1\,\mathrm{\mu m}$
h shell thickness	$0.06\, r_c$	$15\,\mathrm{nm}$
ρ_s shell mass density	$3.31\, m r_c^{-3}$	$1100\,\mathrm{kg\,m^{-3}}$
ρ_{gas} gas mass density	$3.89 \times 10^{-3}\, m\, r_c^{-3}$	$1.293\,\mathrm{kg\,m^{-3}}$
E_{3D} Young's modulus	$9.96 \times 10^{8}\, k_B T_0\, r_c^{-3}$	$264\,\mathrm{MPa}$
ν Poisson's ratio	0.5	0.5
K_a bulk modulus	$4.42 \times 10^{5}\, k_B T_0\, r_c^{-2}$	$2.93 \times 10^{-2}\,\mathrm{Nm^{-1}}$
μ shear modulus	$1.47 \times 10^{5}\, k_B T_0\, r_c^{-2}$	$9.77 \times 10^{-3}\,\mathrm{Nm^{-1}}$
κ_C bending parameter	$204.3\, k_B T_0$	$8.46 \times 10^{-19}\, J$

As seen in Fig. S10, the resulting velocity profile is linear. The overall similarity between the profile of the undisturbed flow and the profile of the flow in the presence of the GV indicates that the GV does not significantly disrupt the shear flow, implying limited resistance.

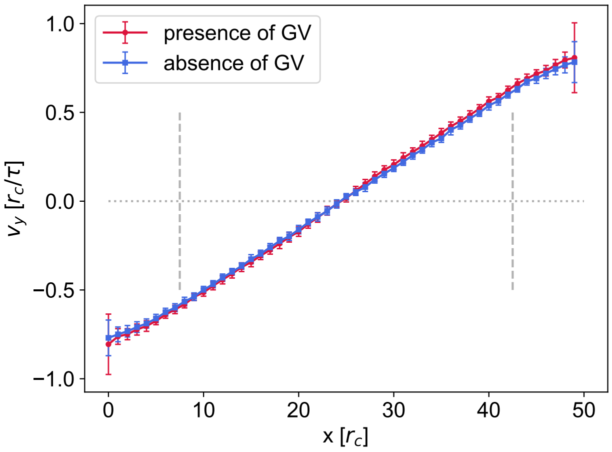

Figure S10: Velocity profile v_y along the x axis of the simulation box at $\dot{\gamma} \sim 0.035\ \tau^{-1}$. Data from a simulation of a GV in water (red points) are compared with simulation data of pure water (blue).

Supplementary References

(1) Boehler, J. P. *Applications of Tensor Functions in Solid Mechanics*; Springer Vienna, 1987; p 13–30.

(2) Evans, E. A.; Skalak, R. *Mechanics and thermodynamics of biomembranes*; CRC-Press, 1980.

(3) Turner, M. J.; Clough, R. W.; Martin, H. C.; Topp, L. Stiffness and deflection analysis of complex structures. *J. Aeronaut. Sci.* **1956**, *23*, 805–823.

(4) Economides, A.; Arampatzis, G.; Alexeev, D.; Litvinov, S.; Amoudruz, L.; Kulakova, L.; Papadimitriou, C.; Koumoutsakos, P. Hierarchical Bayesian Uncertainty Quantification for a Model of the Red Blood Cell. *Phys. Rev. Appl.* **2021**, *15*, 034062.

(5) Lim HW, G.; Wortis, M.; Mukhopadhyay, R. Red Blood Cell Shapes and Shape Transformations: Newtonian Mechanics of a Composite Membrane: Sections 2.1–2.4. *Soft Matter: Lipid Bilayers and Red Blood Cells* **2008**, *4*, 83–139.

(6) Alexeev, D.; Amoudruz, L.; Litvinov, S.; Koumoutsakos, P. Mirheo: high-performance mesoscale simulations for microfluidics. *Comput. Phys. Commun.* **2020**, *254*, 107298.

(7) Bian, X.; Litvinov, S.; Koumoutsakos, P. Bending models of lipid bilayer membranes: Spontaneous curvature and area-difference elasticity. *Comput. Methods Appl. Mech. Eng.* **2020**, *359*, 112758.

(8) Guckenberger, A.; Gekle, S. Theory and algorithms to compute Helfrich bending forces: a review. *J. Phys. Condens. Matter* **2017**, *29*, 203001.

(9) Nelson, D.; Peliti, L. Fluctuations in membranes with crystalline and hexatic order. *Journal de Physique* **1987**, *48*, 1085–1092.

(10) Jülicher, F. The Morphology of Vesicles of Higher Topological Genus: Conformal Degeneracy and Conformal Modes. *Journal de Physique II* **1996**, *6*, 1797–1824.

(11) Gompper, G.; Kroll, D. M. Random Surface Discretizations and the Renormalization of the Bending Rigidity. *Journal de Physique I* **1996**, *6*, 1305–1320.

(12) Meyer, M.; Desbrun, M.; Schröder, P.; Barr, A. H. *Visualization and Mathematics III*; Springer Berlin Heidelberg, 2003; p 35–57.

(13) Fedosov, D. A. *Multiscale modeling of blood flow and soft matter*; Brown University, 2010.

(14) Zoelly, R. *Ueber ein Knickungsproblem an der Kugelschale*; Buchdr. Zürcher & Furrer, 1915.

(15) Chen, S.; Phan-Thien, N.; Khoo, B. C.; Fan, X. J. Flow around spheres by dissipative particle dynamics. *Phys. Fluids* **2006**, *18*, 103605.

(16) Yaghoubi, S.; Shirani, E.; Pishevar, A. R.; Afshar, Y. New modified weight function for the dissipative force in the DPD method to increase the Schmidt number. *EPL* **2015**, *110*, 24002.

(17) Fedosov, D. A.; Caswell, B.; Popel, A. S.; Karniadakis, G. E. Blood flow and cell-free layer in microvessels. *Microcirculation* **2010**, *17*, 615–628.

(18) Pan, W.; Fedosov, D. A.; Caswell, B.; Karniadakis, G. E. Predicting dynamics and rheology of blood flow: A comparative study of multiscale and low-dimensional models of red blood cells. *Microvasc. Res.* **2011**, *82*, 163–170.

(19) Fedosov, D. A.; Pan, W.; Caswell, B.; Gompper, G.; Karniadakis, G. E. Predicting human blood viscosity in silico. *Proc. Natl. Acad. Sci. U.S.A.* **2011**, *108*, 11772–11777.

(20) Groot, R. D.; Warren, P. B. Dissipative Particle Dynamics: Bridging the Gap between Atomistic and Mesoscopic Simulation. *J. Chem. Phys.* **1997**, *107*, 4423–4435.

(21) Zhang, S.; Huang, A.; Bar-Zion, A.; Wang, J.; Mena, O. V.; Shapiro, M. G.; Friend, J. The vibration behavior of sub-micrometer gas vesicles in response to acoustic excitation determined via laser doppler vibrometry. *Adv. Funct. Mater.* **2020**, *30*, 2000239.

(22) Ye, Z. On sound scattering and attenuation of Albunex® bubbles. *J. Acoust. Soc. Am.* **1996**, *100*, 2011–2028.

(23) Marmottant, P.; Bouakaz, A.; Jong, N. d.; Quilliet, C. Buckling resistance of solid shell bubbles under ultrasound. *J. Acoust. Soc. Am.* **2011**, *129*, 1231–1239.
